# Supplementary material for: A Video- and Case-Based Curriculum on the Management of Alcohol Use Disorder for Internal Medicine Residents
Source: MedEdPORTAL. 2022 Mar 31;18:11236. doi: 10.15766/mep_2374-8265.11236 (PMC8967922; doi:10.15766/mep_2374-8265.11236)
Supplement: Supplementary file 1 — Session 1 Learner Guide.docxSession 1 Facilitator Guide.docxSession 1 Concept Video.mp4Session 2 Learner Guide.docxSession 2 Facilitator Guide.docxSession 2 Concept Video.mp4Session 3 Learner Guide.docxSession 3 Facilitator Guide.docxPre- and Postsurvey Tool.docxFaculty Survey.docx [file mep_2374-8265.11236-s001.zip › A. Session 1 Learner Guide.docx]

**Cases for Discussion: Introduction to Alcohol Use Disorder Curriculum**

*Welcome to the* ***first*** *in a 3-part series on management of alcohol use disorder!*

***Agenda:***

- ***First 15 minutes: watch video and complete the video guide***
- ***Last 15 minutes: read case and answer quick questions***
- ***Take home SBIRT review***

**Video Guide**

1. What are the ASAM levels of treatment for alcohol use disorder and what kinds of patient factors determine which level of care is most appropriate?

| Intensity |  |
| --- | --- |
| LEVEL 0.5 |  |
| Level 1 |  |
| Level 2 |  |
| Level 3 |  |
| Level 4 |  |

1. Please complete the table.

|  | Abstinence-based? | Spiritual or secular? | Guiding principle |
| --- | --- | --- | --- |
| Women for sobriety |  |  |  |
| LifeRing |  |  |  |
| SMART  Recovery |  |  |  |
| Moderation Management |  |  | n/a |

1. What are some markers of high-quality addiction treatment programs (according to SAMSHA and the NIAAA)?

a.

b.

c.

d.

e.

**Discussion Guide**

*This is a de-identified and simplified note from a resident patient. Please read about him. Then, we’ll talk about him as a group.*

61-year-old male veteran presenting for routine follow-up

Active Problems:

1. Hyperlipidemia - on rosuvastatin 20 mg

2. Alcohol use - last 3 out of 4 urine drug screens notable for alcohol and cannabis.

3. Steatohepatitis - on RUQ U/S 10 months ago; AST:ALT 2:1 pattern

4. Chronic back pain - on gabapentin & voltaren, tramadol stopped due to ongoing alcohol use

5. Hypertension

6. Peripheral neuropathy - c/b recurrent foot infections. RPR and HIV negative, A1C 5.4.

7. Folic acid/vitamin D/magnesium/potassium deficiency - likely 2/2 EtOH

8. chronic tachycardia: TSH normal, sinus rhythm, likely 2/2 alcohol use

HPI:

# EtOH/EtOH liver disease: He states he is cutting down. Has been hard to stop altogether and he still states he enjoys alcohol, but he feels motivated to cut down. Still getting strong “urges” to drink and sometimes has more than he means to. Drank 6 beers over the past 8 days. Willing to have labs checked today. Does not want to pursue AA or addiction treatment at this point. He would consider medications. He understands the danger of continuing to drink. No history of alcohol withdrawal. In the past was drinking 8-10 drinks 3-4 times per week. Some marijuana.

OBJECTIVE

Vitals: Temp 98.6; Pulse 88; RR: 20; BP 129/75; BMI 26

Normal physical exam. No tremor, stigmata of chronic liver disease, diaphoresis, or jaundice.

**Does this patient sound familiar or like other patients you have cared for, in either the inpatient or outpatient settings? What concerns might you have about a patient like this with alcohol use disorder?**

**This patient has been reducing his alcohol use over time. How do you assess the risk of severe withdrawal?**

**Risk factors for severe withdrawal**

**This patient is reluctant to get involved with AA. What is the structure of AA? What are possible barriers to involvement in AA?**

**Screening, Brief Intervention, and Alcoholics Anonymous (AA)**

**Definitions**

“A Standard Drink” is **14 g** of alcohol. Generally, this translates to:

- 12 oz beer
- 5 oz wine
- 4 oz fortified wine or sherry
- 1.5 oz liquor (gin, vodka, whiskey)
- *Note: these are rough estimates. Beer, for example, can range from 5-13% alcohol by volume, which obviously changes how big a standard drink is*

Other ways to measure alcohol:

- A case of beer: 24 cans of beer
- Bottle of wine: 6-12 drinks
- A pint of liquor: 8 ½ drinks
- A “fifth” of liquor: 17 drinks

Dietary guidelines for Americans for low-risk alcohol use:

|  | Average drinks per week  (____ or fewer) | No more than ___ drinks in 1 day |
| --- | --- | --- |
| Women | 7 | 3 |
| Men | 14 | 4 |
| *Everyone: no alcohol when it is dangerous, contraindicated, or socially unacceptable (ie. while driving, while at most places of employment, while on certain medications, while legally underage)* | | |

“Binge day:” drinking more than 4 drinks in a day for women or men 65 years or older, or more than 5 drinks in a day for men under 65

“Heavy Drinking:” More than 5 “binge days” per month

**Screening Recommendation**

USPSTF recommendation: screen for unhealthy alcohol use in primary care settings in adults 18 years or older, including pregnant women, and provide persons engaged in risky or hazardous drinking with brief behavioral counseling interventions to reduce unhealthy alcohol use

**Screening Tools**

| AUDIT-C | 0 | 1 | 2 | 3 | 4 |
| --- | --- | --- | --- | --- | --- |
| How often do you have a drink containing alcohol? | Never | Monthly or less | 2-4 times per month | 2-3 times per week | 4 or more times per week |
| How many standard drinks containing alcohol do you have on a typical day of drinking? | 1-2 | 3-4 | 5-6 | 7-9 | 10 or more |
| How often do you have 6 or more drinks on 1 occasion? | Never | Less than monthly | Monthly | Weekly | Daily or almost daily |

*Score of 5 or more will trigger AUDIT-C clinical reminder at the VA*

**Single Item Screening Question (SASQ)**

**Diagnosis**

Alcohol Use Disorder is diagnosed when at least **2** of **11** criteria are met:

| Category | Criteria |
| --- | --- |
| Impaired Control | Alcohol is taken in larger amounts or over a longer period than intended |
|  | Persistent desire or unsuccessful efforts to cut down or control alcohol use |
|  | A great deal of time is spent in activities necessary to obtain alcohol, drink alcohol, or recover from the effects of alcohol |
|  | Craving or a strong desire/urge to use alcohol |
| Social Impairment | Recurrent alcohol use resulting in failure to fulfill major obligations at work, home or school |
|  | Recurrent use despite having persistent or recurrent interpersonal or social problems caused or exacerbated by alcohol use |
|  | Important social, recreational, or occupational activities are reduced or given up because of alcohol use |
| Risky Use | Recurrent alcohol use in situations in which it is physically hazardous |
|  | Alcohol use is continued despite knowledge of having a persistent or recurrent physical or psychological problem that is likely caused or exacerbated by alcohol use |
| Pharmacologic Criteria | Tolerance (increasing amounts of alcohol needed to achieve the same effect, or diminished effect with continued use of the same amount of alcohol) |
|  | Withdrawal (characteristic withdrawal syndrome or alcohol is consumed to relieve or avoid withdrawal symptoms) |
| Mild: 2-3 criteria; Moderate: 4-5 criteria; Severe: 6 or more criteria | |

**Brief Intervention Tools**

| **Steps: 5 As** | **OARS Skills:** with examples |
| --- | --- |
| **Ask:** ask about alcohol use, and ask for permission to talk about screening results  **Advise:** May a recommendation about low risk alcohol use  **Assess:** Readiness to change and presence of change talk  **Assist**: Discuss options for change and make a plan about next steps  **Arrange*:*** follow-up and referral if indicated | **Open-ended questions:** How might your life be different without alcohol in it?  **Affirmations:** Thanks for working so hard to track your drinking!  **Reflections:** It sounds like your worried that your drinking is making your depression worse.  **Summary statement:** So, it sounds like you’re not sure your drinking is a big deal, and things are going well at work. At the same time, your wife is concerned about your drinking. You’ve also noticed that you’ve been drinking more recently. Did I get that right? |

Citations:

1. Bush K, Kivlaban DR, McDonell MB, Fihn SD, Bradley KA. “The AUDIT alcohol consumption questions (AUDIT-C): an effective brief screening test for problem drinking." *Arch Intern Med* 1998;158(16):1789-95.
2. Centers for Disease Control and Prevention. Alcohol Related Disease Impact (ARDI) application, 2013. Available at [www.cdc.gov/ARDI](https://nccd.cdc.gov/DPH_ARDI)
3. Edelman EJ, Fiellin DA. “Alcohol use.” *Annal Int Med*. 2016;164(1): 1-16.
4. Mee-Lee D, Shulman GD. “The ASAM criteria and matching patients to treatment.” In: The ASAM Essentials of Addiction Medicine, 3^rd^ edition. Philadelphia: Wolters Kluwer; 2020. Pages 172-178.
5. Moos RH, Moos BS. Participation in treatment and Alcoholics Anonymous: a 16-year follow-up of initially untreated individuals. *J Clin Psychol*. 2006;62(6):735–750. doi:10.1002/jclp.20259
6. NIAAA Alcohol Treatment Navigator. “How to spot quality treatment.” Accessed 1/26/2020. Available: <https://alcoholtreatment.niaaa.nih.gov/>
7. O’Connor EA, Perdue LA, Senger CA, et al. Screening and Behavioral Counseling Interventions to Reduce Unhealthy Alcohol Use in Adolescents and Adults: Updated Evidence Report and Systematic Review for the US Preventive Services Task Force. JAMA. 2018;320(18):1910–1928.
8. Smith PC, Schmidt SM, Allensworth-Davies D, Saitz R. Primary care validation of a single-question alcohol screening test [published correction appears in J Gen Intern Med. 2010 Apr;25(4):375]. *J Gen Intern Med*. 2009;24(7):783-788.
9. U.S. Department of Health and Human Services and U.S. Department of Agriculture. 2015–2020 Dietary. Guidelines for Americans.
