# Supplementary material for: A Video- and Case-Based Curriculum on the Management of Alcohol Use Disorder for Internal Medicine Residents
Source: MedEdPORTAL. 2022 Mar 31;18:11236. doi: 10.15766/mep_2374-8265.11236 (PMC8967922; doi:10.15766/mep_2374-8265.11236)
Supplement: Supplementary file 1 — Session 1 Learner Guide.docxSession 1 Facilitator Guide.docxSession 1 Concept Video.mp4Session 2 Learner Guide.docxSession 2 Facilitator Guide.docxSession 2 Concept Video.mp4Session 3 Learner Guide.docxSession 3 Facilitator Guide.docxPre- and Postsurvey Tool.docxFaculty Survey.docx [file mep_2374-8265.11236-s001.zip › B. Session 1 Facilitator Guide.docx]

**Cases for Discussion: Introduction to Alcohol Use Disorder Curriculum**

*Welcome to the* ***first*** *in a 3-part series on management of alcohol use disorder!*

***Instructions for facilitators:***

***Agenda:***

- ***15 minutes: show video and ask residents to complete the video guide***
- ***15 minutes: read case and answer discussion questions***
- ***Hand out SBIRT review/resource sheet for residents***

**Video Guide:**

1. What are the ASAM levels of treatment for alcohol use disorder and what kinds of patient factors determine which level of care is most appropriate?

| Intensity |  |
| --- | --- |
| LEVEL 0.5 | Brief intervention |
| Level 1 | Outpatient: less than 9 hours/wk |
| Level 2 | Intensive OP (9-20 hours/wk)  Partial hospitalization (>20 hours/wk) |
| Level 3 | Residential |
| Level 4 | Medically monitored withdrawal management |

ASAM recommends looking at 6 patient factors to determine appropriate level of care, including history of relapse, risk for withdrawal, readiness to change, living situation, medical comorbidities, and psychiatric/mental health comorbidities. Ultimately, addiction specialists are often the ones who determine the appropriate level of care for a patient- but when a patient is struggling in outpatient treatment, it is often an indication that he or she needs a higher level of care,

1. Please complete the table.

|  | Abstinence-based? | Spiritual or secular? | Guiding principle |
| --- | --- | --- | --- |
| Women for sobriety | Yes | Mostly secular | 13 affirmations |
| LifeRing | Yes | Secular | 3 S model |
| SMART  Recovery | Mostly, but moderation goals ok too | Secular | 4 points |
| Moderation Management | No- but many people eventually need to pursue an abstinence goal | Secular | None |

1. What are some markers of high-quality addiction treatment programs (according to SAMSHA and the NIAAA)?

Both SAMHSA (substance abuse and mental health services administration) and the NIAAA (national institute on alcohol abuse and alcoholism) have some suggestions about what to look for in a treatment program. In the video, we talked about NIAAA’s recommendations. Both agree that looking at accreditation and credentials can be important (both programs and providers can be credentialed). Both also emphasize the importance of evidence-based treatment modalities (like those listed in question 3) and establishing plans for long-term relapse prevention and continuing care. NIAAA recommends looking for programs that also include comprehensive assessment and personalized treatment plans. SAMHSA recommends programs that include medication treatment and family programming.

*This is a de-identified and simplified note from a resident patient. Please read about him. Then, we’ll talk about him as a group.*

61-year-old male veteran presenting for routine follow-up

Active Problems:

1. Hyperlipidemia - on rosuvastatin 20 mg

2. Alcohol use - last 3 out of 4 urine drug screens notable for alcohol and cannabis.

3. Steatohepatitis - on RUQ U/S 10 months ago; AST:ALT 2:1 pattern

4. Chronic back pain - on gabapentin & voltaren, tramadol stopped due to ongoing alcohol use

5. Hypertension

6. Peripheral neuropathy - c/b recurrent foot infections. RPR and HIV negative, A1C 5.4.

7. Folic acid/vitamin D/magnesium/potassium deficiency - likely 2/2 EtOH

8. chronic tachycardia: TSH normal, sinus rhythm, likely 2/2 alcohol use

HPI:

# EtOH/EtOH liver disease: He states he is cutting down. Has been hard to stop altogether and he still states he enjoys alcohol, but he feels motivated to cut down. Still getting strong “urges” to drink and sometimes has more than he means to. Drank 6 beers over the past 8 days. Willing to have labs checked today. Does not want to pursue AA or addiction treatment at this point. He would consider medications. He understands the danger of continuing to drink. No history of alcohol withdrawal. In the past was drinking 8-10 drinks 3-4 times per week. Some marijuana.

OBJECTIVE

Vitals: Temp 98.6; Pulse 88; RR: 20; BP 129/75; BMI 26

Normal physical exam. No tremor, stigmata of chronic liver disease, diaphoresis, or jaundice.

**Does this patient sound familiar or like other patients you have cared for, in either the inpatient or outpatient settings? What concerns might you have about a patient like this with alcohol use disorder?**

This is an open-ended question meant to illicit any questions or concerns that the learners have. There are no right or wrong answers. Some people may feel overwhelmed by a patient with ongoing active alcohol use disorder. Some may be excited, since it sounds like he is cutting down already on his alcohol use.

I hope that this does sound familiar to people. Unfortunately, alcohol use disorder is common- about 8% of the US adult population meets criteria for alcohol use disorder, and alcohol use was associated with 88,000 deaths in the US between 2006 and 2010 (CDC ARDI calculator).

**This patient has been reducing his alcohol use over time. How do you assess the risk of severe withdrawal?**

**Risk factors for severe withdrawal**

History of withdrawal or delirium tremens

Concurrent benzodiazepines

>100 g alcohol/day (~7 drinks)

Evidence of withdrawal on exam

**This patient is reluctant to get involved with AA. What is the structure of AA? What are possible barriers to involvement in AA?**

AA is a mutual support group for people struggling with alcohol use disorder. There are meetings a variety of times in almost every city in the country. Mutual support groups are **peer-to-peer organizations** without clinically trained facilitators. Instead, mutual support groups draw on a wealth of personal experiences with alcohol use disorder. Meetings occur in person and online and take different forms.

**Meeting Terminology:**

***Step meetings:*** focused on one of the 12 steps of recovery

***Speaker meetings:*** one speaker shares details of his or her journey with AUD and recovery

***Discussion meetings:*** members share thoughts on a theme (ie. Gratitude, forgiveness, etc)

All three meeting types can be either ***open*** or ***closed***

***Open:*** times and locations are publicly available, and anyone can attend

***Closed:*** participants must be invited to attend (many people with sensitive community jobs).

While AA has traditionally been difficult to study, there is some evidence that it is helpful. One study showed that people who participate in AA programing have better long-term outcomes in terms of abstinence and social function even 16 years out from treatment, and AA attendance is thought to lead to about 3.3 more days of abstinence every month (Moos 2006).

The resident guide includes a review of screening, brief intervention, and alcoholics anonymous.

Citations

1. Bush K, Kivlaban DR, McDonell MB, Fihn SD, Bradley KA. “The AUDIT alcohol consumption questions (AUDIT-C): an effective brief screening test for problem drinking." *Arch Intern Med* 1998; 158(16): 1789-95.
2. Centers for Disease Control and Prevention. Alcohol Related Disease Impact (ARDI) application, 2013. Available at [www.cdc.gov/ARDI](http://www.cdc.gov/ARDI)
3. Edelman EJ, Fiellin DA. “Alcohol use.” *Annal Int Med*. 2016; 164(1): 1-16.
4. Mee-Lee D, Shulman GD. “The ASAM criteria and matching patients to treatment.” In: The ASAM Essentials of Addiction Medicine, 3^rd^ edition. Philadelphia: Wolters Kluwer; 2020. Pages 172-178.
5. Moos RH, Moos BS. Participation in treatment and Alcoholics Anonymous: a 16-year follow-up of initially untreated individuals. *J Clin Psychol*. 2006;62(6):735–750. doi:10.1002/jclp.20259
6. NIAAA Alcohol Treatment Navigator. “How to spot quality treatment.” Accessed 1/26/2020. Available: <https://alcoholtreatment.niaaa.nih.gov/>
7. O’Connor EA, Perdue LA, Senger CA, et al. Screening and Behavioral Counseling Interventions to Reduce Unhealthy Alcohol Use in Adolescents and Adults: Updated Evidence Report and Systematic Review for the US Preventive Services Task Force. JAMA. 2018;320(18):1910–1928.
8. Smith PC, Schmidt SM, Allensworth-Davies D, Saitz R. Primary care validation of a single-question alcohol screening test [published correction appears in J Gen Intern Med. 2010 Apr;25(4):375]. *J Gen Intern Med*. 2009;24(7):783-788.
9. U.S. Department of Health and Human Services and U.S. Department of Agriculture. 2015–2020 Dietary. Guidelines for Americans
