# Supplementary material for: A Video- and Case-Based Curriculum on the Management of Alcohol Use Disorder for Internal Medicine Residents
Source: MedEdPORTAL. 2022 Mar 31;18:11236. doi: 10.15766/mep_2374-8265.11236 (PMC8967922; doi:10.15766/mep_2374-8265.11236)
Supplement: Supplementary file 1 — Session 1 Learner Guide.docxSession 1 Facilitator Guide.docxSession 1 Concept Video.mp4Session 2 Learner Guide.docxSession 2 Facilitator Guide.docxSession 2 Concept Video.mp4Session 3 Learner Guide.docxSession 3 Facilitator Guide.docxPre- and Postsurvey Tool.docxFaculty Survey.docx [file mep_2374-8265.11236-s001.zip › D. Session 2 Learner Guide.docx]

**Case for Discussion: Psychosocial Supports for Alcohol Use Disorder**

*Welcome to the* ***second*** *in a 3-part series on management of alcohol use disorder!*

***Agenda:***

- ***First 15 minutes: read case as a group, then discuss questions.***
- ***Second 15 minutes: watch video on medications for AUD***

Theresa is a 29-year-old army veteran with a history of PTSD, depression, tobacco use, and alcohol use disorder. She has been lost to follow-up for the past couple of years and is coming in because she has had 2 positive pregnancy tests at home. You confirm that she is indeed pregnant. While she isn’t sure how she will care for another baby, she says she wants to continue the pregnancy.

Theresa tells you that she usually drinks a bottle and a half of wine (750 mL per bottle) throughout the day. She does use any other illicit substances or prescription medications. She is currently not working after being laid off. She has a 2-year-old son at home and is his primary caregiver. She and her boyfriend recently broke up. Without his income, she is afraid she won’t be able to pay her rent next month. She was able to significantly reduce her drinking during her last pregnancy, but with all these stressors, her alcohol use has been going up and she feels like she is out of control. She is worried that her drinking might have an impact on her health and her baby’s health. She is asking for your help.

*What kinds of clinical services are available to help Theresa with her alcohol use disorder? Is there any more information you might help you decide about the most appropriate level of service for her?*

*What level of treatment is most appropriate for Theresa right now? What are the indications for medically managed detoxification?*

**Indications for medically managed detoxification**

Theresa undergoes medically supervised withdrawal and she and her inpatient team believe she might benefit from a residential treatment program.

*What advice exists for patients when evaluating possible treatment programs? What if she weren’t a veteran?*

| Feature | Explanation |
| --- | --- |
|  | Has the program been accredited by the joint commission or CARF (commission on accreditation of rehabilitation facilities)?  Does the center employ therapists and physicians with special training in addiction?  Accreditation is *not the same* as licensure- all centers must be licensed by the state, but the bar for licensure is much lower than the bar for accreditation. |
|  | Will the program address Theresa’s medical needs (prenatal care)?  Will the program address her psychiatric needs (PTSD/depression)?  Will the program address housing needs, vocational needs, spiritual needs? |
|  | Does the plan change based on the findings of the comprehensive assessment?  *She may need more (or less) time depending on her progress* |
|  | Does the program offer CBT, motivational enhancement, or 12-step facilitation?  Does the program offer medications? |
|  | How does the program facilitate a plan for ongoing support when a patient “graduates” to a less intensive treatment level? |

Theresa is interested in a high-quality program. While she thinks a neighbor might be able to watch her son for a couple of days, she doesn’t think she would be able to go to a residential care facility long-term due to her childcare responsibilities. She doesn’t want to let her son down.

*How might you respond to Theresa’s concerns?*

She follows with high risk obstetrics for care during her pregnancy. After her treatment, she asks you about AA. She wants to be involved with other people who are working on their alcohol use, and she wants to continue to be abstinent from alcohol. However, she has some concerns about the spiritual focus of AA.

*What other kinds of support groups are available for Theresa?*

Citations:

1. Edelman EJ, Fielin DA. In the clinic: Alcohol Use. *Annals of Internal Medicine.* 2016;164(1):ITC 1-16. DOI: 10.7326/AITC201601050
2. Milligan K, Niccols A, Sword W, Thabane L, Henderson J, Smith A, Liu J. Maternal substance use and integrated treatment programs for women with substance abuse issues and their children: a meta-analysis. *Subst Abuse Treat Prev Policy.* 2010;5(21). DOI:[10.1186/1747-597X-5-21](https://dx.doi.org/10.1186%2F1747-597X-5-21).
3. Mee-Lee D, Shulman GD. “The ASAM criteria and matching patients to treatment.” In: The ASAM Essentials of Addiction Medicine, 3^rd^ edition. Wolters Kluwer; 2020:172-178.
4. NIAAA Alcohol Treatment Navigator. “How to spot quality treatment.” Accessed 1/26/2020. Available: <https://alcoholtreatment.niaaa.nih.gov/>
5. Tsutsumi S, Timko C, Zemore SE. Ambivalent attendees: transitions in group affiliation among those who choose a 12-step alternative for addiction. *Addictive behaviors.* 2020;102. DOI: 10.1016/j.addbeh.2019.106143.
6. Zemore SE, Lui C, Mericle A, Hemberg J, Kaskutas LA. A longitudinal study of the comparative efficacy of Women for Sobriety, LifeRing, SMART recovery, and 12-step groups for those with AUD. *J Subst Abuse Treat.* 2018;88:18-26.

**Session 2 Video Guide**

| **Medication** | **Indications** | **Contraindications** | **Side effects** | **Dose** | **Lab monitoring** |
| --- | --- | --- | --- | --- | --- |
| Naltrexone |  |  |  |  |  |
| Acamprosate |  |  |  |  |  |
| Topiramate |  |  |  |  |  |
| Disulfiram |  |  |  |  |  |
