# Supplementary material for: A Video- and Case-Based Curriculum on the Management of Alcohol Use Disorder for Internal Medicine Residents
Source: MedEdPORTAL. 2022 Mar 31;18:11236. doi: 10.15766/mep_2374-8265.11236 (PMC8967922; doi:10.15766/mep_2374-8265.11236)
Supplement: Supplementary file 1 — Session 1 Learner Guide.docxSession 1 Facilitator Guide.docxSession 1 Concept Video.mp4Session 2 Learner Guide.docxSession 2 Facilitator Guide.docxSession 2 Concept Video.mp4Session 3 Learner Guide.docxSession 3 Facilitator Guide.docxPre- and Postsurvey Tool.docxFaculty Survey.docx [file mep_2374-8265.11236-s001.zip › E. Session 2 Facilitator Guide.docx]

**Case for Discussion: Psychosocial Supports for Alcohol Use Disorder**

*Welcome to the* ***second*** *in a 3-part series on management of alcohol use disorder!*

***Instructions for facilitators:***

*Each resident should receive a copy of the resident discussion guide for today.*

***Agenda:***

- ***First 15 minutes: read case as a group, then discuss questions.***
- ***Second 15 minutes: watch video on medications for AUD. Residents should work on the video guide while they watch.***
- ***Text in red is not visible to learners.***

Theresa is a 29-year-old army veteran with a history of PTSD, depression, tobacco use, and alcohol use disorder. She has been lost to follow-up for the past couple of years and is coming in because she has had 2 positive pregnancy tests at home. You confirm that she is indeed pregnant. While she isn’t sure how she will care for another baby, she says she wants to continue the pregnancy.

Theresa tells you that she usually drinks a bottle and a half of wine (750 mL per bottle) throughout the day. She does use any other illicit substances or prescription medications. She is currently not working after being laid off. She has a 2-year-old son at home and is his primary caregiver. She and her boyfriend recently broke up. Without his income, she is afraid she won’t be able to pay her rent next month. She was able to significantly reduce her drinking during her last pregnancy, but with all these stressors, her alcohol use has been going up and she feels like she is out of control. She is worried that her drinking might have an impact on her health and her baby’s health. She is asking for your help.

*What kinds of clinical services are available to help Theresa with her alcohol use disorder? Is there any more information you might help you decide about the most appropriate level of service for her?*

| Intensity |  |
| --- | --- |
| LEVEL 0.5 | Brief intervention |
| Level 1 | Outpatient: less than 9 hours/wk |
| Level 2 | Intensive OP (9-20 hours/wk)  Partial hospitalization (>20 hours/wk) |
| Level 3 | Residential |
| Level 4 | Medically monitored withdrawal management |

The American Society for Addiction Medicine (ASAM) recommends evaluating six components when determining the most appropriate level of care (Mee-Lee 2020):

| Risk for withdrawal | Any history of seizures or DTs? When was the last time she went a day without drinking? |
| --- | --- |
| Medical comorbidities | Any history of pregnancy complications? |
| Mental health comorbidities | How are things going with her PTSD and depression?  Any history of sexual trauma or violence that might make it hard for her to be in a mixed gender environment? |
| Relapse history | How long was she able to reduce her drinking after her last pregnancy? |
| Living environment | (given in prompt) |

*What level of treatment is most appropriate for Theresa right now? What are the indications for medically managed detoxification?*

Theresa has multiple indications to start her treatment in a **medically monitored setting**:

1. Pregnancy: Normal physiologic changes in pregnancy, like tachycardia, can mask early withdrawal symptoms, and withdrawal during pregnancy can be particularly dangerous (Edelman 2016). Alcohol use during pregnancy has a significant impact on fetal development, so achieving abstinence quickly is a priority.
2. Elevated risk of severe withdrawal: Finally, a bottle of wine has ~6 standard drinks in it, so she is at increased risk of severe withdrawal (>100 g alcohol per day; she is having about 9 glasses of wine per night, which is about 126 g of ethanol)
3. Relative indications: unstable housing and poor social supports

**Indications for medically managed detoxification**

Elevated risk for severe withdrawal

Medical indication for inpatient care (pancreatitis, acute hepatitis)

Psychiatric indication for inpatient care (suicidality, psychosis)

Pregnancy

.

Theresa undergoes medically supervised withdrawal and she and her inpatient team believe she might benefit from a residential treatment program.

*What advice exists for patients when evaluating possible treatment programs?*

It can be hard for patients to find treatment programs, and even harder to find programs that insurance will pay for. As a veteran, Theresa does have access to evidence-based addiction services through the VA.

| Feature | Explanation |
| --- | --- |
| Accreditation | Has the program been accredited by the joint commission or CARF (commission on accreditation of rehabilitation facilities)?  Does the center employ therapists and physicians with special training in addiction?  Accreditation is *not the same* as licensure- all centers must be licensed by the state, but the bar for licensure is much lower than the bar for accreditation. |
| Comprehensive Assessment | Will the program address Theresa’s medical needs (prenatal care)?  Will the program address her psychiatric needs (PTSD/depression)?  Will the program address housing needs, vocational needs, spiritual needs? |
| Personalized Plan | Does the plan change based on the findings of the comprehensive assessment?  *She may need more (or less) time depending on her progress* |
| Evidence-based treatment | Does the program offer CBT, motivational enhancement, or 12-step facilitation?  Does the program offer medications? |
| Ongoing recovery support | How does the program facilitate a plan for ongoing support when a patient “graduates” to a less intensive treatment level? |

While many people have had success in recovery through programs that don’t have all 5 elements of high-quality treatment, programs with more of these markers of quality are likely to be more reputable than programs with few of these markers.

Theresa is interested in a high-quality program. While she thinks a neighbor might be able to watch her son for a couple of days, she doesn’t think she would be able to go to a residential care facility long-term due to her childcare responsibilities. She doesn’t want to let her son down.

*How might you respond to Theresa’s concerns?*

1. Motivational interviewing: what does she identify as a priority?
2. Discuss programs specifically designed for pregnant and parenting mothers:

- Often include prenatal care, employment training, mental health care, parenting classes, and resources for women experiencing intimate partner violence
- Evidence shows these types of programs are effective, though they may not be *more* effective than traditional treatment programs (Milligan 2010).
- May be cost prohibitive

She follows with high risk obstetrics for care during her pregnancy. After her treatment, she asks you about AA. She wants to be involved with other people who are working on their alcohol use, and she wants to continue to be abstinent from alcohol. However, she has some concerns about the spiritual focus of AA.

*What other kinds of support groups are available for Theresa? What is the evidence surrounding these alternate mutual support groups?*

|  | Abstinence-based? | Spiritual or secular? | Sponsors? | Local Availability? |
| --- | --- | --- | --- | --- |
| Women for sobriety | Yes | Mostly secular | No | Monthly in person meetings |
| LifeRing | Yes | Secular | No | Not in Pittsburgh |
| SMART  Recovery | Mostly, but moderation goals ok too | Secular | No | Wednesday/Saturday in Pittsburgh |
| Moderation Management | No, but many people eventually need to pursue an abstinence goal | Secular | No | Wednesday in Pittsburgh |
| AA | Yes | Spiritual | Yes | Numerous meetings every day |

*Evidence summary*

- People who are highly engaged in AA alternatives have similar outcomes and similar drop-out rates as people who commit to AA- the important thing is probably to stay engaged with the recovery community (Zemore 2018).
- People who engage with alternative mutual support groups are more likely to switch mutual support organizations after 6 months than those who start with 12 step programing, and most people who switch get involved with 12 step programs.
- People with more severe alcohol use disorder are more likely to switch than people with more moderate disease. Qualitative data suggests that this is because they have greater needs for mutual support and there is greater 12-step group availability than alternative group availability (Tsutsumi 2020).

Citations:

1. Edelman EJ, Fielin DA. In the clinic: Alcohol Use. *Annals of Internal Medicine* 2016; 164(1): ITC 1-16. DOI: 10.7326/AITC201601050
2. Milligan K, Niccols A, Sword W, Thabane L, Henderson J, Smith A, Liu J. Maternal substance use and integrated treatment programs for women with substance abuse issues and their children: a meta-analysis. *Subst Abuse Treat Prev Policy* 2010; 5 (21). DOI: [10.1186/1747-597X-5-21](https://dx.doi.org/10.1186%2F1747-597X-5-21).
3. Mee-Lee D and Shulman GD. “The ASAM criteria and matching patients to treatment.” In: The ASAM Essentials of Addiction Medicine, 3^rd^ edition. Philadelphia: Wolters Kluwer; 2020. Pages 172-178.
4. NIAAA Alcohol Treatment Navigator. “How to spot quality treatment.” Accessed 1/26/2020. Available: <https://alcoholtreatment.niaaa.nih.gov/>
5. Tsutsumi S, Timko C, Zemore SE. Ambivalent attendees: transitions in group affiliation among those who choose a 12-step alternative for addiction. *Addictive behaviors* 2020; 102. DOI: 10.1016/j.addbeh.2019.106143.
6. Zemore SE, Lui C, Mericle A, Hemberg J, Kaskutas LA. A longitudinal study of the comparative efficacy of Women for Sobriety, LifeRing, SMART recovery, and 12-step groups for those with AUD. *J Subst Abuse Treat* 2018; 88: 18-26.

**Session 2 Video guide (residents have blank table)**

| Medication | Impact | Contraindications | Side effects | Dose | Lab monitoring |
| --- | --- | --- | --- | --- | --- |
| Naltrexone  (First line) | Reduces cravings  Reduces alcohol use  Increases abstinence | 1. Decompensated liver disease; LFTs >3-5 ULN 2. (chronic) opioid use in last 10 days 3. Severe renal disease | Nausea/vomiting  Headache  Fatigue  Weight loss | Oral: 50-100 mg daily  IM: 380 mg every 4 weeks | BMP & LFTs at initiation  Repeat at 6 and 12 months  Annual CMP after first year |
| Acamprosate  (First line for liver disease) | Increases abstinence | 1. Renal impairment with GFR <30 | Diarrhea  ? depression? | Typical dose: 666 mg TID  CrCl 30-50:  333 mg TID | Creatinine at initiation  Periodic renal function |
| Topiramate  (off label first/second line) | Increase abstinence  Reduce alcohol use  Reduce cravings | 1. Nexplanon/ oral contraception 2. Hepatic encephalopathy 3. Nephrolithiasis | Paresthesias  Fatigue/ sedation  Teratogenic  Weight loss | Initiation: 25-50 mg daily  Weekly increase by: 25-50 mg/day  Goal: 100 mg BID  Reduce if CrCl <50 | Initiation: creatinine  Periodic renal function |
| Disulfiram | Increase abstinence  Best if administered by a 3^rd^ party  **No alcohol x12 hours or BAL = 0** | 1. Cognitive impairment 2. Severe cardiovascular, renal, pulmonary, or hepatic disease 3. Prior psychotic disorders 4. *Many* drug interactions | Alcohol-disulfiram reaction  Psychosis  Hepatitis  Neuropathy  Headaches  Metallic taste | 250 mg daily | BMP, LFT, ± blood alcohol level at initiation  Repeat monthly for first 3 months for 6 months  Repeat every 6 months indefinitely |
