# Supplementary material for: A Video- and Case-Based Curriculum on the Management of Alcohol Use Disorder for Internal Medicine Residents
Source: MedEdPORTAL. 2022 Mar 31;18:11236. doi: 10.15766/mep_2374-8265.11236 (PMC8967922; doi:10.15766/mep_2374-8265.11236)
Supplement: Supplementary file 1 — Session 1 Learner Guide.docxSession 1 Facilitator Guide.docxSession 1 Concept Video.mp4Session 2 Learner Guide.docxSession 2 Facilitator Guide.docxSession 2 Concept Video.mp4Session 3 Learner Guide.docxSession 3 Facilitator Guide.docxPre- and Postsurvey Tool.docxFaculty Survey.docx [file mep_2374-8265.11236-s001.zip › G. Session 3 Learner Guide.docx]

**Cases for Discussion: AUD Management Session 3**

*Welcome to the* ***final*** *part of our 3-part series on management of alcohol use disorder!*

***Agenda:***

- ***15 minutes: Medication vignettes***
- ***10 minutes: Go through conclusion to the case from session 1 and answer questions.***
- ***5 minutes: Reflection worksheet***

**Case Vignettes**

What medication would be most appropriate for the patients described below? Are there any medications that would be *contraindicated or inappropriate* for these patients? Unless otherwise specified, assume all patients below are engaged in some form of addiction-focused psychosocial support (regular mutual support group attendance, individual therapy, or alcohol treatment program) and have been diagnosed with moderate or severe alcohol use disorder.

1. **48-year-old man with a history recent hospitalization for hepatic encephalopathy and advanced liver disease**
2. **27-year-old woman without significant past medical history; uses combined oral contraceptive pills for contraception**
3. **51-year-old highly motivated man with a history of opiate use disorder on daily methadone maintenance administered 6 days a week through methadone clinic**
4. **31-year-old woman who struggles to take any of her medications regularly and has not had success with other pharmacologic interventions in the past**
5. **29-year-old man who is interested in reducing his drinking, but doesn’t want to stop drinking completely**
6. **61-year old man with stage 4/5 CKD (chronic kidney disease)**

**Return to Case from Session 1**

*This is a de-identified (and somewhat simplified/lightly edited) note from a real patient followed in resident clinic. Please read about him. Then, we’ll talk about him as a group.*

61-year-old male veteran presenting for routine follow-up

Active Problems:

1. Alcohol use - last 3 out of 4 urine drug screens notable for alcohol and cannabis.

2. Steatohepatitis - on RUQ U/S 10 months ago; AST:ALT 2:1 pattern

3. Chronic back pain

4. Hypertension

5. Peripheral neuropathy 2/2 alcohol use

HPI:

# EtOH/EtOH liver disease: He states he is cutting down. Has been hard to stop altogether and he still states he enjoys alcohol, but he feels motivated to cut down. Still getting strong “urges” to drink and sometimes has more than he means to. Drank 6 beers over the past 8 days. Does not want to pursue AA or specialty addiction treatment at this point. He would consider medications. He understands the danger of continuing to drink.

OBJECTIVE: vitals and physical exam unremarkable

*This patient states he is interested in medications, but not ready to engage with mutual support groups or addiction focused care. How would you approach medication prescribing for him?*

*You decide to prescribe him a medication. What medication would you choose? What testing would you to obtain?*

Six Months Later:

Veteran continues to use alcohol, though overall significant improvement since starting naltrexone. Is drinking even less now (maybe once a week). Still having intermittent spikes in ast and ggt. Is taking naltrexone every day and pharmacy fills back this up. He does not want to pursue AA @ this point.

*How long should you continue his naltrexone?*

**Reflection worksheet**

**One thing I learned about managing patients with alcohol use disorder during these 3 sessions is….**

**One thing I would like to try doing in a future encounter with a patient with alcohol use disorder is…**

**What questions do you still have about referral to treatment and medication for patients with alcohol use disorder?**

**(If you choose to include your email I will try to answer the question if I can!)**

**Answers to Homework from Session 2**

| Medication | Indications | Contraindications | Side effects | Dose | Lab monitoring |
| --- | --- | --- | --- | --- | --- |
| Naltrexone | Moderate to severe AUD  Reduces cravings  Goal of abstinence  Goal of reducing drinking | Severe, decompensated liver disease  (chronic) opioid use in last 10 days  Not studied in severe renal disease | Nausea/vomiting  Headache  Fatigue  Weight loss | Oral: 50 mg daily  (can increase to 100 daily)  IM: 380 mg every 4 weeks | BMP, LFTs at initiation  Repeat at 6 and 12 months  Annual CMP after first year |
| Acamprosate | Moderate to severe AUD  Goal of abstinence | Renal impairment with GFR <30 | Diarrhea  ? possible increased risk of depression or suicidal ideation | Typical dose: 666 mg TID  CrCl 30-50:  333 mg TID | BMP (before to establish renal function)  Periodically (once every year or two) |
| Topiramate | Moderate to severe AUD (***off label use)***  Goal of abstinence  Goal of reducing drinking  Reduce cravings | Interacts with nexplanon and oral contraceptives (IUDs are ok)  Can cause confusion- use care in patients with a hx of hepatic encephalopathy | Paresthesias  Fatigue/ sedation  Teratogenic  Weight loss | Start at 25 or 50 mg per day  increase dose once a week by 50 mg per day  Max dose:  100 mg BID | Check renal function- may need to dose reduce if renal function impaired |
| Disulfiram | Moderate to severe AUD  Goal of abstinence  Best if therapy can be administered by a 3^rd^ party  **Must have no drink x12 hours or BAL = 0** | Cognitive impairment  Severe cardiovascular, renal, pulmonary, or hepatic disease  Prior psychotic disorders  *Many* drug interactions  No alcohol in food, mouthwash, OTCs | Alcohol-disulfiram reaction  Psychosis  Hepatitis  Neuropathy  Headaches  Metallic taste | 250 mg daily | BMP, LFT, ± blood alcohol level at initiation  Repeat monthly for first 3 months  Repeat every 6 months after that |
