# Supplementary material for: A Video- and Case-Based Curriculum on the Management of Alcohol Use Disorder for Internal Medicine Residents
Source: MedEdPORTAL. 2022 Mar 31;18:11236. doi: 10.15766/mep_2374-8265.11236 (PMC8967922; doi:10.15766/mep_2374-8265.11236)
Supplement: Supplementary file 1 — Session 1 Learner Guide.docxSession 1 Facilitator Guide.docxSession 1 Concept Video.mp4Session 2 Learner Guide.docxSession 2 Facilitator Guide.docxSession 2 Concept Video.mp4Session 3 Learner Guide.docxSession 3 Facilitator Guide.docxPre- and Postsurvey Tool.docxFaculty Survey.docx [file mep_2374-8265.11236-s001.zip › H. Session 3 Facilitator Guide.docx]

**Cases for Discussion: AUD Management Session 3**

*Welcome to the* ***final*** *part of our 3-part series on management of alcohol use disorder!*

***Agenda:***

- ***15 minutes: Medication vignettes***
- ***10 minutes: Go through conclusion to the case from session 1 and answer questions.***
- ***5 minutes: Reflection worksheet***
- ***Text in red is not visible to learners.***

**Case Vignettes**

What medication would be most appropriate for the patients described below? Are there any medications that would be *contraindicated or inappropriate* for these patients? Unless otherwise specified, assume all patients below are engaged in some form of addiction-focused psychosocial support (regular mutual support group attendance, individual therapy, or alcohol treatment program) and have been diagnosed with moderate or severe alcohol use disorder.

1. **48-year-old man with a history recent hospitalization for hepatic encephalopathy and advanced liver disease**

**Best choice**: acamprosate 666 mg TID. It is safe in all stages of liver disease.

**Poor choices:**

- Naltrexone is an option for compensated cirrhosis and child-pugh class A/B but should be avoided in advanced disease.
- Topiramate is not the best option in a patient with a history of hepatic encephalopathy because it can also cause encephalopathy (relative contraindication)

1. **27-year-old woman without significant past medical history; uses combined oral contraceptive pills for contraception**

**Best choice:**

- naltrexone (due to efficacy, once daily dosing at 50 mg, and few side effects.

**Alternates:**

- Acamprosate, though it is limited by TID dosing and may be less effective for helping people reduce alcohol use.

**Poor choice:**

- Topiramate is teratogenic and reduces the efficacy of combined oral contraceptives and may not be the best choice for a patient who is on COCPs. Topiramate can be used with long-acting reversible contraception.

1. **51-year-old highly motivated man with a history of opiate use disorder on daily methadone maintenance administered 6 days a week through methadone clinic**

**Best choices:**

- acamprosate 666 TID (FDA approved)
- topiramate 100 mg BID (not FDA approved but likely effective)

**Alternatives:**

- Because he is already receiving methadone maintenance, some methadone programs would consider daily administration of disulfiram (250 mg daily). Disulfiram is likely to be more effective in this kind of an observed setting than in other settings. Because he has a complex addiction history and is already connected with addiction specialists, most generalists would work together with his team of addiction specialists to address his alcohol use.

**Contraindicated:** Naltrexone would precipitate acute withdrawal and would prevent him from benefitting from methadone.

1. **31-year-old woman who struggles to take any of her medications regularly and has not had success with other pharmacologic interventions in the past**

**Best choices:** IM naltrexone 380 mg every 4 weeks.

While there haven’t been head-to-head efficacy trials for oral vs IM naltrexone for AUD, IM naltrexone may be helpful for patients who have struggled with adherence in the past. Because this woman has struggled with medication therapy and maintenance of recovery in the past, it would also be appropriate to consult with an addiction specialist for assistance.

1. **29-year-old man who is interested in reducing his drinking, but doesn’t want to stop drinking completely**

**Best choices:** Oral naltrexone (50 mg daily) has been shown to reduce cravings and binge drinking in addition to increasing abstinence.

**Alternatives:**

- Topiramate has been shown to reduce binge drinking, though it is not FDA approved. The combination of topiramate and alcohol can intensify the sedating effects of both drugs.

**Less good choices:**

- When studied, acamprosate doesn’t seem to reduce binge drinking, though it is effective for promoting abstinence. However, in clinical practice, it would be reasonable to use acamprosate if the patient was significantly invested in this medication for some reason.

1. **61-year old man with stage 4/5 CKD (chronic kidney disease)**

Patients with advanced kidney disease can be difficult to treat. Acamprosate is contraindicated in advanced kidney disease (GFR <30). Similarly, naltrexone has not been studied in advanced renal disease. Topiramate has to be dose reduced in renal disease. Gabapentin has been used experimentally and is permitted in low doses in renal disease, but the data to support its use is not robust and there are concerns that combining gabapentin and alcohol might increase the side effects of both. It would be reasonable to consult with an addiction specialist.

**Return to Case from Session 1**

*This is a de-identified (and somewhat simplified/lightly edited) note from a real patient followed in resident clinic. Please read about him. Then, we’ll talk about him as a group.*

61-year-old male veteran presenting for routine follow-up

Active Problems:

1. Alcohol use - last 3 out of 4 urine drug screens notable for alcohol and cannabis.

2. Steatohepatitis - on RUQ U/S 10 months ago; AST:ALT 2:1 pattern

3. Chronic back pain

4. Hypertension

5. Peripheral neuropathy 2/2 alcohol use

HPI:

# EtOH/EtOH liver disease: He states he is cutting down. Has been hard to stop altogether and he still states he enjoys alcohol, but he feels motivated to cut down. Still getting strong “urges” to drink and sometimes has more than he means to. Drank 6 beers over the past 8 days. Does not want to pursue AA or CTAD at this point. He would consider medications. He understands the danger of continuing to drink.

OBJECTIVE: vitals and physical exam unremarkable

*This patient states he is interested in medications, but not ready to engage with mutual support groups or addiction focused care. How would you approach medication prescribing for him?*

In general, we would recommend engaging with psychosocial support. However, for a patient who is not ready to engage with psychosocial support or mutual support groups, it is reasonable to start a medication for AUD. This patient would benefit from reducing his alcohol intake, even if he isn’t ready to stop drinking completely. A medication might be particularly helpful for this particular patient, as he has already successfully reduced his alcohol use.

*You decide to prescribe him a medication. What medication would you choose? What testing would you to obtain?*

Acamprosate, topiramate, and naltrexone would all be reasonable. I would start with naltrexone, because it is FDA-approved for this indication and requires daily dosing. Acamprosate has not been shown to reduce alcohol use in people who don’t stop drinking altogether.

This patient has a known history of steatohepatitis and LFT abnormalities. Naltrexone can’t be used in advanced decompensated cirrhosis, but it is safe in a patient like this. Even if this patient did develop cirrhosis, naltrexone is still considered safe in early stages of disease.

VA prescribing guidelines recommend getting a baseline CMP and repeat CMP in 6 months, as naltrexone has been associated with LFT abnormalities in rare cases.

The patient was started on naltrexone.

Six Months Later:

Veteran continues to use alcohol, though overall significant improvement since starting naltrexone. Is drinking even less now (maybe once a week). Still having intermittent spikes in ast and ggt. Is taking naltrexone every day and pharmacy fills back this up. He does not want to pursue AA at this point.

*How long should this patient continue on naltrexone?*

The **first** question to answer is whether this outcome is successful. Though he is still having intermittent spikes in his LFTs, his alcohol use is decreasing significantly. For most people, this would be considered a successful harm reduction outcome.

*Note:* If he hadn’t had any change to his drinking, we could consider *switching* to a medication with a different mechanism of action (such as topiramate) rather than *adding* a second medication, which hasn’t been shown to be effective and may increase side effects.

The **second** question is how long to continue a successful medication. There is a paucity of data about the optimal duration of medication treatment for people with AUD. SAMHSA recommends treating for at least 6-12 months, and states it is reasonable to continue for longer than a year in patients who are benefitting from treatment. In practice, many experts will continue prescribing as long as the patient is benefiting from the medication and not experiencing significant side effects.

**References**

Anton, R. et al. Combined pharmacotherapies and behavioral interventions for alcohol dependence: the COMBINE study: a randomized controlled trial. *JAMA* 2006;295(17): 2003-2017.

Guglielmo R, Martinotti G, Quatrale M, et al. Topiramate in Alcohol Use Disorders: Review and Update. *CNS Drugs*. 2015;29(5):383–395.

Kranzler HR, Soyka M. Diagnosis and pharmacotherapy of alcohol use disorder*.* *JAMA*. 2018;320:815-824.

SAMHSA and NIAAA. *Medication for the treatment of alcohol use disorder: a brief guide.* HHS Publication No. (SMA) 15-4907. SAMHSA 2015.

US department of veterans affairs. *Alcohol use disorder: leading the charge in the treatment of AUD.* Veterans health administration. 2017.

Witkiewitz et al. Maintenance of WHO risk drinking level reductions and posttreatment functioning following a large AUD clinical trial. *Alcoholism: clinical and exp research* 2019;43(5):979-987.

**Reflection worksheet**

**One thing I learned about managing patients with alcohol use disorder is….**

**One thing I would like to try doing in a future encounter with a patient with alcohol use disorder is…**

**What questions do you still have about referral to treatment and medication for patients with alcohol use disorder?**

**(optional: you include your email I will try to answer the question if I can!)**

**Medication Table**

| **Medication** | **Indications** | **Contraindications** | **Side effects** | **Dose** | **Lab monitoring** |
| --- | --- | --- | --- | --- | --- |
| Naltrexone | Moderate to severe AUD  Reduces cravings  Goal of abstinence  Goal of reducing drinking | Severe, decompensated liver disease  (chronic) opioid use in last 10 days  Not studied in severe renal disease | Nausea/vomiting  Headache  Fatigue  Weight loss | Oral: 50 mg daily  (can increase to 100 daily)  IM: 380 mg every 4 weeks | BMP, LFTs at initiation  Repeat at 6 and 12 months  Annual CMP after first year |
| Acamprosate | Moderate to severe AUD  Goal of abstinence | Renal impairment with GFR <30 | Diarrhea  ? possible increased risk of depression or suicidal ideation | Typical dose: 666 mg TID  CrCl 30-50:  333 mg TID | BMP (before to establish renal function)  Periodically (once every year or two) |
| Topiramate | Moderate to severe AUD (***off label use)***  Goal of abstinence  Goal of reducing drinking  Reduce cravings | Interacts with nexplanon and oral contraceptives (IUDs are ok)  Can cause confusion- use care in patients with a hx of hepatic encephalopathy | Paresthesias  Fatigue/ sedation  Teratogenic  Weight loss | Start at 25 or 50 mg per day  increase dose once a week by 50 mg per day  Max dose:  100 mg BID | Check renal function- may need to dose reduce if renal function impaired |
| Disulfiram | Moderate to severe AUD  Goal of abstinence  Best if therapy can be administered by a 3^rd^ party  **Must have no drink x12 hours or BAL = 0** | Cognitive impairment  Severe cardiovascular, renal, pulmonary, or hepatic disease  Prior psychotic disorders  *Many* drug interactions  No alcohol in food, mouthwash, OTCs | Alcohol-disulfiram reaction  Psychosis  Hepatitis  Neuropathy  Headaches  Metallic taste | 250 mg daily | BMP, LFT, ± blood alcohol level at initiation  Repeat monthly for first 3 months  Repeat every 6 months after that |
