# Supplementary material for: A Video- and Case-Based Curriculum on the Management of Alcohol Use Disorder for Internal Medicine Residents
Source: MedEdPORTAL. 2022 Mar 31;18:11236. doi: 10.15766/mep_2374-8265.11236 (PMC8967922; doi:10.15766/mep_2374-8265.11236)
Supplement: Supplementary file 1 — Session 1 Learner Guide.docxSession 1 Facilitator Guide.docxSession 1 Concept Video.mp4Session 2 Learner Guide.docxSession 2 Facilitator Guide.docxSession 2 Concept Video.mp4Session 3 Learner Guide.docxSession 3 Facilitator Guide.docxPre- and Postsurvey Tool.docxFaculty Survey.docx [file mep_2374-8265.11236-s001.zip › J. Faculty Survey.docx]

**Appendix J: Faculty Survey**

*Thank you for your help facilitating the pre-clinic conferences on alcohol use disorder and for taking the time to provide important feedback on this curriculum.*

1. Did you facilitate session 1: psychosocial supports for alcohol use disorder? This session consisted of a brief review of AA and risk factors for severe alcohol withdrawal. The video focused on ASAM levels of care, high quality addiction care, and mutual support groups such as Women for Sobriety, LifeRing, SMARTRecovery, and Moderation Management. (yes/no)
2. Did you facilitate session 2: pharmacotherapy for alcohol use disorder? This session reviewed treatment intensity and mutual support groups in a case about a pregnant woman with alcohol use disorder. The video discussed pharmacotherapy for alcohol use disorder. (yes/no)
3. Did you facilitate session 3: case vignettes and wrap-up? This session discussed selecting the most appropriate pharmacotherapy for alcohol use disorder based on patient comorbidities. Residents were also asked to recall one thing they learned during the curriculum on a reflection worksheet. (yes/no)
4. What comments or feedback do you have for this curriculum? (open-ended)

**If faculty answered “yes” to question number 1, they were asked the following questions;**

*Now, we’re going to ask some questions about your experiences with session 1: psychosocial supports for alcohol use disorder.*

Please rate your level of agreement with the following statements.

|  | Strongly disagree | Disagree | Neither agree nor disagree | Agree | Strongly Agree |
| --- | --- | --- | --- | --- | --- |
| Facilitating this session increased my confidence discussing mutual support groups for alcohol use disorder with trainees. | 1 | 2 | 3 | 4 | 5 |
| Facilitating this session increased my confidence discussing addiction treatment programs for alcohol use disorder with trainees. | 1 | 2 | 3 | 4 | 5 |
| The educational video was easy to use. | 1 | 2 | 3 | 4 | 5 |
| I would be happy to facilitate this session again. | 1 | 2 | 3 | 4 | 5 |

Thirty minutes were allocated for the residents to go through session 1: psycosocial supports for alcohol use disorder. How did you feel about the amount of time you had to cover this material?

1. It was just right
2. It was too long. I didn’t need this much time to cover this material.
3. It was too short. I needed more time to cover the material.

How long did you spend preparing to facilitate session 1: Psychosocial supports for alcohol use disorder.

1. I did not prepare
2. 10 minutes or fewer
3. 11-20 minutes
4. 21-30 minutes
5. 31-40 minutes
6. More than 40 minutes

**If faculty answered “yes” to question number 2, they were asked the following questions;**

*Now, we’re going to ask some questions about your experiences with session 2: pharmacotherapy for alcohol use disorder.*

Please rate your level of agreement with the following statements.

|  | Strongly disagree | Disagree | Neither agree nor disagree | Agree | Strongly Agree |
| --- | --- | --- | --- | --- | --- |
| Facilitating this session increased my confidence discussing pharmacotherapy for alcohol use disorder with trainees. | 1 | 2 | 3 | 4 | 5 |
| Facilitating this session increased my confidence discussing mutual support groups for alcohol use disorder with trainees. | 1 | 2 | 3 | 4 | 5 |
| Facilitating this session increased my confidence discussing addition treatment programs for alcohol use disorder with trainees. | 1 | 2 | 3 | 4 | 5 |
| The educational video was easy to use. | 1 | 2 | 3 | 4 | 5 |
| I would be happy to facilitate this session again. | 1 | 2 | 3 | 4 | 5 |

Thirty minutes were allocated for the residents to go through session 2: pharmacotherapy for alcohol use disorder. How did you feel about the amount of time you had to cover this material?

1. It was just right.
2. It was too long. I didn’t need this much time to cover this material.
3. It was too short. I needed more time to cover the material.

How long did you spend preparing to facilitate session 2: Pharmacotherapy for alcohol use disorder.

1. I did not prepare
2. 10 minutes or fewer
3. 11-20 minutes
4. 21-30 minutes
5. 31-40 minutes
6. More than 40 minutes

**If faculty answered “yes” to question number 3, they were asked the following questions;**

*Now, we’re going to ask some questions about your experiences with session 3: case vignettes and curriculum wrap-up.*

Please rate your level of agreement with the following statements.

|  | Strongly disagree | Disagree | Neither agree nor disagree | Agree | Strongly Agree |
| --- | --- | --- | --- | --- | --- |
| Facilitating this session increased my confidence discussing pharmacotherapy for alcohol use disorder with trainees. | 1 | 2 | 3 | 4 | 5 |
| I would be happy to facilitate this session again. | 1 | 2 | 3 | 4 | 5 |

Thirty minutes were allocated for the residents to go through session 2: pharmacotherapy for alcohol use disorder. How did you feel about the amount of time you had to cover this material?

1. It was just right
2. It was too long. I didn’t need this much time to cover this material.
3. It was too short. I needed more time to cover the material.

How long did you spend preparing to facilitate session 3: Case vignettes and curriculum wrap-up?

1. I did not prepare
2. 10 minutes or fewer
3. 11-20 minutes
4. 21-30 minutes
5. 31-40 minutes
6. More than 40 minutes

Citations:

This survey was developed by the authors and was not adapted from other sources.
