# Supplementary material for: A Video- and Case-Based Curriculum on the Management of Alcohol Use Disorder for Internal Medicine Residents
Source: MedEdPORTAL. 2022 Mar 31;18:11236. doi: 10.15766/mep_2374-8265.11236 (PMC8967922; doi:10.15766/mep_2374-8265.11236)
Supplement: Supplementary file 1 — Session 1 Learner Guide.docxSession 1 Facilitator Guide.docxSession 1 Concept Video.mp4Session 2 Learner Guide.docxSession 2 Facilitator Guide.docxSession 2 Concept Video.mp4Session 3 Learner Guide.docxSession 3 Facilitator Guide.docxPre- and Postsurvey Tool.docxFaculty Survey.docx [file mep_2374-8265.11236-s001.zip › I. Pre- and Postsurvey Tool.docx]

**Appendix I: Pre/Post Survey Tool**

Thank you for taking part in this survey!

*For post-curriculum survey only*, *we will start by asking the following:*

*What sessions did you attend? Please check all that apply.*

1. *Psychosocial support for alcohol use disorder*
2. *Pharmacotherapy for alcohol use disorder*
3. *Resident case discussion session*
4. *I’m not sure*

Please answer the following true/false questions.

1. In clinical trials, people with alcohol use disorder who take naltrexone report that it decreases their craving for alcohol. **(True)**
2. In clinical trials, naltrexone helps people with alcohol use disorder reduce their alcohol intake, even if they aren’t ready to completely abstain from alcohol. **(True)**
3. Oral naltrexone is usually dosed twice per day. **(False)**
4. Naltrexone injections cost less than oral naltrexone formulations. **(False)**
5. In patients who take opioids chronically, naltrexone can be started once they have been off opioids for at least 24 hours. **(False)**
6. The intramuscular form of naltrexone is dosed every four weeks. **(True)**
7. Naltrexone is safe in patients at all stages of liver disease. **(False)**
8. People with alcohol use disorder must not have had any alcohol for at least 14 days before starting naltrexone. **(False)**
9. It is safe to start disulfiram as soon as a patient with alcohol use disorder no longer appears intoxicated. **(False)**
10. The disulfiram-alcohol reaction can be triggered by trace amounts of alcohol that might be found in food or over the counter medications. **(True)**
11. One advantage of disulfiram is that it has few drug-drug interactions. **(False)**
12. Disulfiram is contraindicated in patients with a history of psychosis. **(True)**
13. Acamprosate is dosed once a day. **(False)**
14. Acamprosate is safe in patients at all stages of liver disease. **(True)**
15. When studied in clinical trials, acamprosate helps patients abstain from alcohol. **(True)**
16. When studied in clinical trials, acamprosate helps patients reduce their drinking, even if they don’t stop drinking completely. **(False)**
17. Acamprosate has few drug-drug interactions. **(True)**
18. In clinical trials, patients with alcohol use disorder benefit most from the combination of acamprosate and naltrexone to promote alcohol abstinence. **(False)**
19. Acamprosate is safe at all stages of renal disease. **(False)**
20. Topiramate is FDA approved for the treatment of alcohol use disorder. **(False)**
21. Topiramate reduces the efficacy of oral contraceptives. **(True)**
22. Topiramate can cause weight loss. **(True)**
23. AA (alcoholics anonymous) is the most widespread mutual support group for alcohol use disorder. **(True)**
24. LifeRing and SMART recovery are mutual support groups without any religious or spiritual background. **(True)**
25. Most people in SMART recovery are interested in abstaining from alcohol completely, but the organization also supports people whose goal is to reduce alcohol use. **(True)**
26. In order to treat patients, alcohol treatment facilities must be accredited by addiction organizations. **(False)**
27. Twelve step facilitation, motivational enhancement therapy, and cognitive behavioral therapy are evidence-based modalities for the treatment of alcohol use disorder. **(True)**
28. According to NIAAA (national institute of alcohol abuse and alcoholism), one marker of a high-quality rehabilitation program is a universal treatment duration, such as a 28-day or 90-day program. **(False)**
29. According to ASAM (American Society of Addiction Medicine), people who are diagnosed with alcohol use disorder should be treated at residential facilities. **(False)**
30. FMLA (family medical leave act) cannot be used to enable patients to attend residential treatment for alcohol use disorder. **(False)**

Next, we’re going to ask some questions about your experiences working with patients with alcohol use disorder. Consider your experiences in both inpatient and outpatient settings. Please rate your level of agreement with the following statements.^1^

|  | | Strongly Disagree | | Disagree | | Somewhat disagree | | Neutral | | Somewhat agree | | Agree | | Strongly Agree |
| --- | --- | --- | --- | --- | --- | --- | --- | --- | --- | --- | --- | --- | --- | --- |
| 1. I feel I have a working knowledge of alcohol and alcohol-related problems. | | 1 | | 2 | | 3 | | 4 | | 5 | | 6 | | 7 |
| 2. I feel I know how to counsel people with alcohol use disorder over the long-term.* | | 1 | | 2 | | 3 | | 4 | | 5 | | 6 | | 7 |
| 3. I feel I can appropriately advise my patients about drinking and its effects. | | 1 | | 2 | | 3 | | 4 | | 5 | | 6 | | 7 |
|  | Strongly disagree | | Disagree | | Somewhat disagree | | Neutral | | Somewhat agree | | Agree | | Strongly agree | |
| 4. I feel I have the right to ask patients about their drinking when necessary. | 1 | | 2 | | 3 | | 4 | | 5 | | 6 | | 7 | |
| 5. If I felt the need, I could easily find someone who would be able to help me formulate the best approach for helping someone with alcohol use disorder.* | 1 | | 2 | | 3 | | 4 | | 5 | | 6 | | 7 | |
| 6. I am interested in the nature of alcohol-related problems and the responses that can be made to them. | 1 | | 2 | | 3 | | 4 | | 5 | | 6 | | 7 | |
| 7. I want to work with people with alcohol use disorder.* | 1 | | 2 | | 3 | | 4 | | 5 | | 6 | | 7 | |
| 8. All in all, I am inclined to feel I am a failure with people with alcohol use disorder.* | 1 | | 2 | | 3 | | 4 | | 5 | | 6 | | 7 | |

|  | Strongly disagree | Disagree | Somewhat disagree | Neutral | Somewhat agree | Agree | Strongly agree |
| --- | --- | --- | --- | --- | --- | --- | --- |
| 9. I feel I do not have much to be proud of when working with people with alcohol use disorder.* | 1 | 2 | 3 | 4 | 5 | 6 | 7 |
| 10. In general, it is rewarding to work with people with alcohol use disorder.* | 1 | 2 | 3 | 4 | 5 | 6 | 7 |
| 11. In general, I like people with alcohol use disorder.* | 1 | 2 | 3 | 4 | 5 | 6 | 7 |

*Indicate places where we changed the term “drinker” to “people with alcohol use disorder. Adapted from Venkat et al.

|  | Strongly disagree | Disagree | Somewhat disagree | Neutral | Somewhat agree | Agree | Strongly agree |
| --- | --- | --- | --- | --- | --- | --- | --- |
| 12. I feel confident working with people with alcohol use disorder | 1 | 2 | 3 | 4 | 5 | 6 | 7 |
| 13. I am confident counseling patients about mutual support groups. | 1 | 2 | 3 | 4 | 5 | 6 | 7 |
| 14. I am confident prescribing medications for alcohol use disorder. | 1 | 2 | 3 | 4 | 5 | 6 | 7 |
| 15. I can counsel patients with alcohol use disorder about evidence-based psychosocial support. | 1 | 2 | 3 | 4 | 5 | 6 | 7 |

Section 3: Demographic and Practice-Based Information:

Finally, we’re going to ask some questions about you.

1. In the last 6 months, how many patients have you treated for alcohol-use disorder or for an alcohol-related complication, such as alcoholic cardiomyopathy, alcoholic liver disease, alcohol withdrawal, accidental injury in the setting of alcohol use, aspiration from alcohol use, alcoholic hepatitis, acute pancreatitis, or similar? Consider both inpatient and outpatient rotations.
2. none
3. 1-5
4. 6-10
5. 11-20
6. 21 or more
7. In the last 6 months, how often have you *referred* a patient to treatment for alcohol use disorder?
8. never
9. 1-5 times
10. 6-10 times
11. 11-20 times
12. 21 or more times
13. In the past 6 months, how often have you *offered* medications to a patient to assist in the treatment of alcohol use disorder?
14. never
15. 1-5 times
16. 6-10 times
17. 11-20 times
18. 21 or more times
19. In the past 6 months, how often have you *prescribed* medications to a patient to assist in the treatment of alcohol use disorder?
20. never
21. 1-5 times
22. 6-10 times
23. 11-20 times
24. 21 or more times
25. What is your clinical year?
    1. PGY-1
    2. PGY-2
    3. PGY-3
26. What is your clinic site?
    1. Veterans affairs
    2. Shadyside
    3. Montefiore
    4. South Hills
27. What degree do you hold?
    1. MD
    2. DO
    3. MBBS
28. What are your plans following residency?
    1. Primary care
    2. Hospitalist
    3. Subspecialty fellowship
    4. General internal medicine fellowship
    5. Not sure
    6. Other

If other, please share your post-graduation plans: ___________

**Citations:**

1 . Venkat A, Aldridge A, Kearney S, et al. Derivation of a Shortened Research Instrument for Measuring Alcohol and Other Drug Atttudes in a Screening, Brief Intervention, and Referral to Treatment (SBIRT) Training Program. *Journal of Science, Humanities, and Arts- JOSHA*. 2017;4(2).

Author adapted attitudes questions to reflect person-centered language.
