# Supplementary material for: A Video- and Case-Based Curriculum on the Management of Alcohol Use Disorder for Internal Medicine Residents
Source: MedEdPORTAL. 2022 Mar 31;18:11236. doi: 10.15766/mep_2374-8265.11236 (PMC8967922; doi:10.15766/mep_2374-8265.11236)
Supplement: Supplementary file 1 — Session 1 Learner Guide.docxSession 1 Facilitator Guide.docxSession 1 Concept Video.mp4Session 2 Learner Guide.docxSession 2 Facilitator Guide.docxSession 2 Concept Video.mp4Session 3 Learner Guide.docxSession 3 Facilitator Guide.docxPre- and Postsurvey Tool.docxFaculty Survey.docx [file mep_2374-8265.11236-s001.zip › Educational_Summary_Report_11236.pdf]

MedEdPORTAL® The AAMC Journal of Teaching and Learning Resources

Original Publication

Open Access

A Video- and Case-Based Curriculum on the Management of Alcohol Use Disorder for Internal Medicine Residents

Sara A. Spinella, MD, MS*, Diana Samberg, MD, MS, Melissa McNeil, MD, MPH, Scott D. Rothenberger, PhD, Payel Jhoom Roy, MD, MSc, Andrea E. Carter, MD, MS

*Corresponding author: sas545@pitt.edu

Abstract

Introduction: Alcohol use disorder (AUD) is commonly undertreated. Physicians cite discomfort with AUD medication as a barrier to treatment. While several curricula teach and assess screening and brief interventions, few teach and assess learner knowledge of treatment options. **Methods:** We created a video- and case-based curriculum for internal medicine residents delivered by 16 internal medicine faculty in three 30-minute sessions at four clinic sites. Learner knowledge, attitudes, and confidence were assessed before and after the curriculum. We used qualitative methods to evaluate learner reflections. We also assessed faculty satisfaction with the curriculum. **Results:** Of 153 residents receiving the curriculum, 35 (23%) completed both pre- and postsurveys. Median percent correct on knowledge questions improved from 67% pre- to 80% postcurriculum ($p < .001$). Confidence increased for all three items assessing it, with a notable increase in confidence with pharmacotherapy (2.9 pre- vs. 4.5 postcurriculum on a 7-point Likert scale with high scores indicating greater confidence, $p < .001$). Positive attitudes toward people with AUD increased from 3.4 pre- to 3.9 postcurriculum ($p < .001$) on a 7-point Likert scale. Learners continued to express concerns about prescribing logistics, the role of primary care, and management of ongoing use. Thirteen of 16 faculty (83%) completed the postcurricular survey; all said they would be happy to facilitate again. **Discussion:** Implementation of this curriculum for the management of AUD improved resident knowledge, attitudes, and confidence in AUD treatment. The curriculum was acceptable to faculty and is ideal for programs looking to expand teaching about AUD.

Keywords
Substance Use, Alcohol Use, Pharmacotherapy, Substance Abuse/Addiction, Internal Medicine, Case-Based Learning

Educational Objectives

By the end of this activity, learners will be able to:

1. Compare and contrast mutual support group options for management of alcohol use disorder (AUD).
2. Describe the levels of care and the types of services offered in clinical addiction treatment programs.
3. Choose an appropriate medication for chronic management of AUD based on patient goals and comorbidities.
4. Describe monitoring parameters for patients on medications for AUD and discuss markers of effective treatment.

Citation:
Spinella SA, Samberg D, McNeil M, Rothenberger SD, Roy PJ, Carter AE. A video- and case-based curriculum on the management of alcohol use disorder for internal medicine residents. *MedEdPORTAL*. 2022;18:11236. https://doi.org/10.15766/mep_2374-8265.11236

Introduction

Alcohol use disorder (AUD) is a common, chronic, relapsing condition characterized by significant psychological, physical, and social distress. There are almost 100,000 alcohol-attributable deaths annually within the United States, with an average of 29 years of life lost among people with AUD.[1] AUD is treatable with mutual support groups (MSGs), psychosocial counseling, and medication for AUD (MAUD). However, patients with AUD are underreferred for treatment, with only 8% of adults with active AUD receiving addiction counseling or MAUD.[2] Qualitative studies suggest that many people with AUD would be interested in medication[3] and may be more willing to engage in MAUD through primary care than through specialty care.[4] However, even fewer patients are offered pharmacotherapy in primary care than are offered counseling.[5,6] Nationally, physicians report that they do not prescribe MAUD because of skepticism about efficacy, lack of provider knowledge,[7] and reluctance to continue MAUD started by other providers.[8] Addiction specialists believe that physician education about existing

Copyright © 2022 Spinella et al. This is an open-access publication distributed under the terms of the Creative Commons Attribution license.

medications is the most important next step to increase MAUD prescribing.[9]

Review of published curricula reveals a relative paucity of curricula on treatment of AUD. Many curricula that address alcohol use focus on screening and brief intervention rather than management of AUD, and these topics are covered elsewhere within our residency.[10-16] Available curricula that do teach about MAUD require 5-16 hours of learner time and do not specifically assess learner outcomes related to MAUD.[17-19] One recent curriculum specifically addressing medications for both opioid use disorder and AUD was well received by learners, but its impact on resident knowledge is unknown.[20] To our knowledge, there are no published curricula that include material on MSG alternatives to Alcoholics Anonymous (AA).

To address these curricular gaps, we developed a three-part curriculum to teach internal medicine (IM) residents how to prescribe MAUD and counsel patients about MSGs and addiction treatment programs. Although we evaluated our curriculum within our IM residency program, due to immediately available curricular time, the management of AUD is shared with multiple other specialties, including family medicine and psychiatry, so this curriculum may be of interest to those programs as well. The curriculum requires 90 minutes total of curricular time and incorporates videos and case-based discussion spread over 3 months using evidence-based educational strategies to enhance learners' retention of the content.

Methods

Setting and Participants

General IM faculty delivered this curriculum to categorical IM residents at four resident continuity clinic sites at an urban academic medical center between March 2020 and June 2020. Our residency was a hospital-based program with a focus on both general IM and preparation for subspecialization; a majority of residents in our program went on to complete additional fellowship training after graduation.

The curriculum was delivered during three preexisting 30-minute preclinic conference sessions. Faculty were accustomed to teaching short sessions based on standardized curricula during this time and received curricular material by email 1 week before sessions were scheduled. Faculty also had the opportunity to attend an optional 1-hour grand rounds lecture on management of AUD in the primary care setting prior to facilitating these sessions.

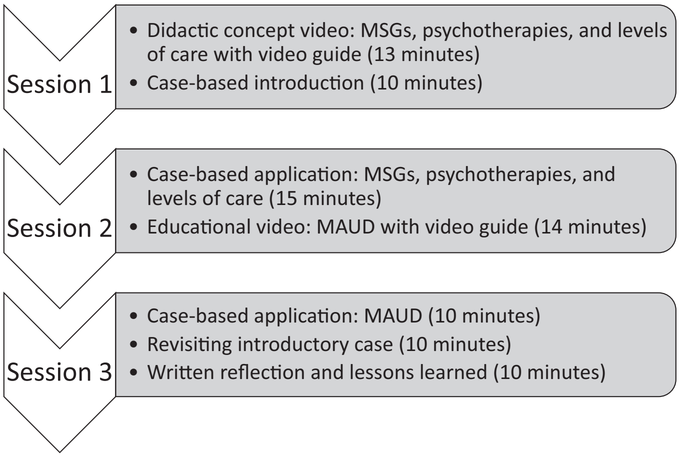

Figure 1. Curricular design: The curriculum involved three 30-minute sessions. Sessions 2 and 3 asked learners to apply material from prior sessions. Abbreviations: MAUD, medication for alcohol use disorder; MSGs, mutual support groups.

To deliver the curriculum, all faculty had access to a computer with a screen visible to small resident groups of four to 10 learners.

This project was supported by the Office of Academic Affiliations Advanced Fellowship in Women's Health at the Pittsburgh Veterans Affairs (VA) Healthcare System, and statistical analysis was funded by the University of Pittsburgh Division of General Internal Medicine. The project was deemed not research (educational evaluation) by the University of Pittsburgh and VA Pittsburgh Institutional Review Boards.

Curriculum Design

We developed three 30-minute sessions to teach IM residents how to manage outpatients with AUD (Figure 1). Our curricular content was based on national treatment guidelines and reviewed by local content experts. Faculty received all curricular materials by email in advance of the sessions.

Session 1 introduced psychosocial treatment for AUD using a structured session guide (Appendix A: resident guide, Appendix B: faculty guide). The session opened with a 13-minute didactic concept video (Appendix C) that introduced MSGs, psychotherapy, levels of care, and markers of rehabilitation quality. To promote active engagement, residents were asked to complete a worksheet based on the didactic content (Appendix A) while watching the video. In the second 15 minutes, residents and preceptors discussed an exemplary case of a veteran with AUD and several alcohol-related comorbidities. This case elicited common challenges in caring for people with AUD in the ambulatory setting, including emotional responses

Copyright © 2022 Spinella et al. This is an open-access publication distributed under the terms of the Creative Commons Attribution license.

to managing addiction and medical concerns about advising abstinence in patients at risk for withdrawal. This session concluded with a discussion about AA. At the end of the session, residents received a self-study handout reviewing screening, brief intervention, and motivational interviewing skills; these topics were covered in depth elsewhere in the residency curriculum.

Session 2 began with a case-based discussion in which learners applied material from the first session about MSGs and psychotherapy to a case about a pregnant veteran with AUD (Appendix D: resident guide, Appendix E: faculty guide). Questions in this case emphasized indications for inpatient detoxification, considerations in choosing residential treatment programs, and barriers to treatment, including cost and parenting responsibilities. The case ended with a comparison between AA and alternative MSGs. The second half of the session was devoted to a 14-minute concept video (Appendix F) that introduced learners to MAUD options. The video focused on FDA-approved medications, naltrexone, acamprosate, and disulfiram, but also presented data on off-label use of topiramate for managing cravings. Facilitators provided learners with a blank table on MAUD options to complete while watching the video.

Session 3 opened with a series of case vignettes that required learners to choose the most appropriate MAUD based on patient goals and comorbidities ((Appendix G: resident guide, Appendix H: faculty guide). Then, learners revisited the patient case from Session 1 and discussed a pharmacologic treatment plan. This case was used to generate a discussion about defining treatment success, reinforcing appropriate monitoring, and considering duration of pharmacologic treatment. Finally, residents were asked to provide written short-answer reflections on lessons learned during the curriculum and to set a goal about applying these lessons in future patient encounters.

Sessions 1 and 2 were delivered 1-3 weeks apart, and sessions 2 and 3 were delivered 5-7 weeks apart, depending on clinical site. The curriculum utilized evidence-based educational techniques to enhance retention, including spaced learning,[21] retrieval practice,[22] active learning,[23] and goal setting.[24]

Evaluation

We assessed the impact of our curriculum on residents' knowledge, confidence, and attitudes using a survey administered both pre- and postcurriculum (Appendix I). To measure knowledge, we developed 30 novel true/false items (22 questions on MAUD, five questions on counseling, and three questions on MSGs). We chose a true/false format because of other evaluation tools using this format, including an assessment of knowledge about naltrexone[25] and the previously validated Student Alcohol Questionnaire designed to measure college students' knowledge regarding alcohol.[26] To measure confidence, we developed four novel 7-point Likert-scale items (1 = *strongly disagree,* 7 = *strongly agree*). To measure attitudes, we adapted the previously validated Survey of Attitudes and Perceptions (SAP),[27] which included 11 items rated on a 7-point Likert scale (1 = *strongly disagree,* 7 = *strongly agree*). We made adaptations only to reflect person-first language, with references to "drinkers" changed to "people with alcohol use disorder." This survey was reviewed by local content experts to ensure accuracy and breadth of content and was pilot tested with recent IM residency graduates for clarity and to determine the amount of time needed for survey completion. The survey was administered electronically via REDCap. Learners completed the precurriculum survey during the first 10 minutes of session 1 and the postcurriculum survey 1-3 weeks after session 3. To improve response rate, residents received 10 minutes of protected time and two email reminders to complete the postcurriculum survey.

We assessed faculty experiences with delivering the curriculum via a survey 1 week after the final session (Appendix J). Faculty were asked if they would be "happy to facilitate the session again" on a 5-point Likert scale (1 = *strongly disagree,* 5 = *strongly agree*). They were also asked whether facilitating each session changed their confidence in teaching these topics. Finally, faculty were asked how long they needed to prepare for each session. This survey was administered electronically via REDCap, with one reminder email sent to all faculty who had led at least one session.

We qualitatively assessed the impact of the curriculum on residents' future practice and future directions through an open-ended written-response reflection handed out and collected at the end of the final session. Residents were asked (1) one thing they learned, (2) one thing they would like to try in a future encounter, and (3) what questions they still had about management of AUD.

Data Analysis

Data analysis was performed using STATA 16.0 (StataCorp). All residents who completed the pre- and postsurveys were included in the analysis of knowledge, confidence, and attitudes. We compared median percent correct on the knowledge items pre- and postcurriculum using the Wilcoxon signed rank test. We compared the mean composite Likert scores for confidence and attitudes pre- and postcurriculum using paired Student *t* tests. Descriptive statistics were used to report faculty facilitator

Copyright © 2022 Spinella et al. This is an open-access publication distributed under the terms of the Creative Commons Attribution license.

responses. Qualitative themes from the residents' open-ended written responses were identified by two reviewers (Sara A. Spinella and Diana Samberg), and all reflections were categorized by theme. Discrepancies were resolved by consensus.

Results

Participant Characteristics

The curriculum was delivered to 153 categorical IM residents across four clinical sites. Of these, 97 (64%) completed the pretest, and 53 (35%) completed the posttest; 35 (23%) completed both the pre- and posttests and were included in our analysis. Our sample featured broad representation of all clinical years and a variety of postgraduation career plans among residents included in the final analysis (Table 1). Three of four clinical sites were represented in our sample; only six of 153 residents had clinic at the site that did not provide any data. Of the 35 residents included in the analysis, the majority reported being present for at least two sessions, although nine (26%) attended just one session and two (6%) reported they did not attend any sessions. Residents who missed sessions were not expected to make them up. The pre- and posttests were emailed to the entire residency class regardless of session attendance because session attendance was not monitored.

Curricular Impact

Median percent correct on the 30-question true/false knowledge items improved from 67% (interquartile range [IQR]: 60%-73%) precurriculum to 80% postcurriculum (IQR: 70%-83%; $p < .001$; Figure 2). There was a significant improvement in the 22 MAUD questions ($p < .001$), while there was no significant change in the three questions about MSGs ($p = 1.00$) and five questions about psychosocial treatments ($p = .133$). The scores of all four confidence questions were combined to form a composite mean confidence score, which improved from 3.7 ($SD = 0.94$) precurriculum to 4.9 ($SD = 0.76$) postcurriculum on the 7-point Likert scale ($p < .001$; Figure 2). Notably, we observed the most dramatic improvement in confidence about MAUD, which went from 2.8 ($SD = 1.30$) to 4.5 ($SD = 1.30$; $p < .001$). Attitude questions were also combined to form a composite mean attitude score, which improved from 4.5 ($SD = 0.85$) pre- to 5.1 ($SD = 0.69$) postcurriculum on the 7 point-Likert scale ($p < .001$; Figure 2).

Table 1. Resident Demographics ($N = 35$)[a]

Characteristic	No. (%)
Level of training	
PGY 1	15 (43)
PGY 2	13 (37)
PGY 3	7 (20)
Postgraduate career plans	
Primary care	5 (14)
Hospitalist	1 (3)
Subspecialty fellowship	21 (60)
General medicine fellowship	4 (11)
Unsure	4 (11)
No. sessions attended	
0	2 (6)
1	9 (26)
2	11 (31)
3	13 (37)

[a]Only residents who completed surveys both before and after the curriculum were included in the analysis.

Qualitative Analysis

In an open-ended reflection on one learning point gained from the curriculum, residents identified learning around all curricular domains except clinical psychosocial addiction treatment. Residents set goals about prescribing MAUD, counseling on MAUD, counseling on MSGs, and eliciting or understanding patient-centered goals for treatment. Residents' reflections on ongoing areas of uncertainty were most diverse; many residents asked questions about prescribing logistics, the role of primary care versus specialty addiction services, and management of relapse or nonabstinence (Table 2).

Faculty Experiences With the Curriculum

Of the 16 faculty who delivered at least one session, 13 (81%) completed the facilitator survey. Four faculty facilitated one session, three facilitated two sessions, and six facilitated all three sessions. All faculty agreed or strongly agreed they would be happy to facilitate again for all three sessions. The majority of faculty needed less than 10 minutes to prepare for each session. All faculty who facilitated sessions 2 or 3, which addressed MAUD, reported that facilitating the session increased their confidence discussing pharmacotherapy with trainees. In contrast, faculty were divided about whether sessions that addressed MSGs increased their confidence discussing MSGs with trainees.

Discussion

AUD is a common and debilitating disease that is frequently diagnosed in the primary care setting, yet many generalists feel uncomfortable with MAUD prescribing or referrals to treatment programs or MSGs, and few curricula teach this content. To address this gap, we developed a case- and video-based curriculum for IM residents with a focus on MAUD, psychosocial treatment options, and the range of available MSGs. Our curriculum improved resident knowledge, attitudes, and confidence about management of AUD and was highly acceptable to faculty facilitators. Qualitatively, residents identified

Copyright © 2022 Spinella et al. This is an open-access publication distributed under the terms of the Creative Commons Attribution license.

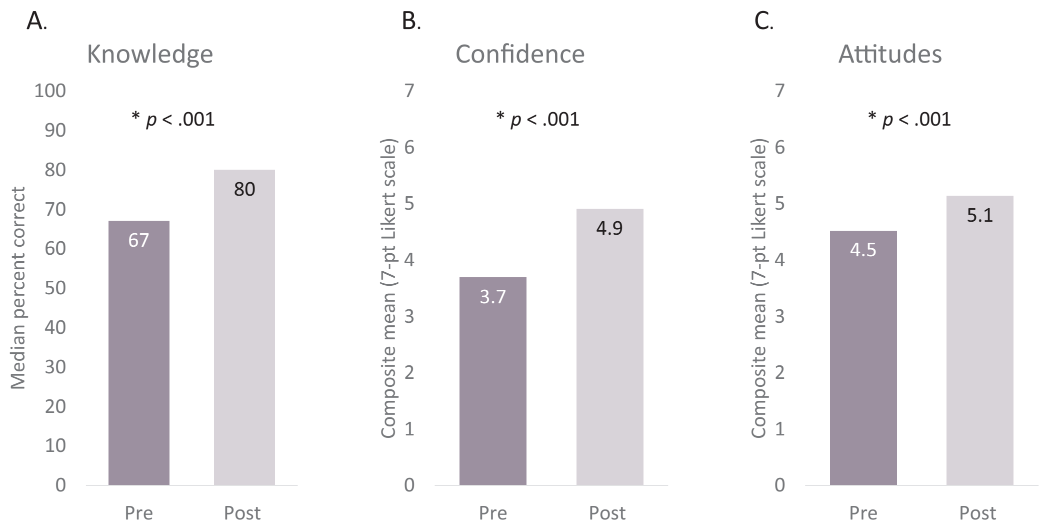

Figure 2. Impact of curriculum on resident knowledge, confidence, and attitudes. A: Median percent correct on 30-item true/false test pre- and postcurriculum. B: The scores of all four confidence questions were combined to form a composite mean confidence score pre- and postcurriculum. Confidence was measured on a 7-point Likert scale where higher scores indicated greater confidence. C: Attitude questions were also combined to a composite mean attitude score, which was compared pre- and postcurriculum.

knowledge gains about MSGs and MAUD and reflected that elicitation of patient goals was a key feature of this intervention.

This is the first curriculum that demonstrates an impact on knowledge about MAUD. The deliberate inclusion of adult learning principles such as spaced learning and retrieval practice was designed to increase learner retention and ensure that a large proportion of our residents were exposed to the material, even if they were absent for any given session. We also used short periods of preexisting protected curricular time to prevent

Table 2. Qualitative Themes and Example Responses[a]

Theme	Representative Responses
What did you learn during this curriculum?	
MAUD: side effects and contraindications	"There is an [intramuscular] formulation of naltrexone, naltrexone requires LFT monitoring, and acamprosate should be avoided in [chronic kidney disease] 4/5."
MAUD: goals of treatment	"Using naltrexone or topiramate for patients who want to cut back but not stop."
MAUD: general information	"Learning all 4 of medication options for treatment; was previously only aware of naltrexone."
Mutual support groups	"The different support groups and characteristics, ie. Secular or religious and who can participate. This has helped me better prepare how to counsel patients."
What is one thing you will try in a future encounter?	
Prescribe or counsel about MAUD	"I rarely discuss medication options—usually just counsel(ing). Will start looking for opportunities to prescribe medications that could increase success."
	"Talk about medication options—I have not been as good about this as I am with tobacco cessation meds."
Counsel about mutual support groups	"Promote AA and other options like non-religious groups."
Elicit or understand goals	"Ask if a patient wants to cut back on alcohol if they don't want to quit outright."
What questions do you still have about AUD management?	
Logistics and site-specific questions	"Is there a way to refer patients for treatment outside the [Veterans Administration]?"
Role of primary care	"When should I consider addiction medicine consult? After how much time on meds?"
	"This should be done by social work. [Doctors] don't have time for this other than placing a referral."
Specifics about MAUD	"Should medications be universal?"
	"How often do you need LFTs for naltrexone? What is the threshold to stop if LFTs are elevated?"
Communication skills	"How to navigate when a patient would seem to benefit from [specialty addiction treatment services] but are refusing to go."
Management of relapse or nonabstinence	"Whether to continue or do a new trial in a patient who is non-adherent to meds."
Treatment duration	"Rates of adherence to different meds, especially if more than daily, and long term care if successful abstinence—temporarily continued or taper off?"

Abbreviations: AA, Alcoholics Anonymous; AUD, alcohol use disorder; LFT, liver function test; MAUD, medication for alcohol use disorder.
[a]Residents were asked to reflect on their learning, goals for future encounters, and remaining questions. We identified key themes and quotes that exemplified these themes.

Copyright © 2022 Spinella et al. This is an open-access publication distributed under the terms of the Creative Commons Attribution license.

competing demands on resident time without needing resident coverage or additional faculty teaching time.

Our approach has strengths that may facilitate broader curricular dissemination. We used guided videos to standardize the teaching of key content and to enable general IM faculty to facilitate sessions with minimal preparation. Additionally, standardized didactics were intended to address concerns faculty may have had about their own knowledge and skills regarding MAUD, given nationally low rates of MAUD prescribing in primary care.[5,6] The fact that faculty did not require additional training or significant preparation time for this curriculum was essential to making it a feasible intervention. We coupled didactic videos with guided, case-based discussions to give learners the opportunity to apply material to challenging cases reflecting resident clinic populations.

Our study also had several limitations. Although our curriculum was delivered at multiple clinical sites, including both hospital-based academic clinics and VA clinics, all residents came from a single residency program, and our sample size was small and was further limited by response rate. The response rate to our pre- and posttests was relatively low. Furthermore, only a minority of learners were able to attend all three sessions. Our curriculum was designed such that key content was repeated at multiple sessions to improve retention, but learners who attended only one or two sessions likely did not benefit from this design. Additionally, two residents who completed the survey reported they did not attend any sessions; they may have reviewed the materials sent out via email asynchronously, although we did not ask this specifically in our postsurvey. Due to the small sample size, we were unable to assess whether future career plans or clinic site affected efficacy. While the SAP was a previously validated measure of attitudes toward people with AUD, validation of the knowledge and confidence assessments was limited to review by content experts and brief pilot testing for clarity. The small number of questions about MSGs and psychosocial support limited our ability to evaluate knowledge change in those domains. Similarly, our faculty survey was not previously validated.

Interestingly, our residents received a median of 68% correct on the preintervention knowledge survey, despite reporting a lack of confidence about MAUD prescribing. The true/false format was chosen to reflect other assessments of knowledge about alcohol use in the literature,[25,26] but it had limited sensitivity for detecting change because residents were expected to receive 50% correct by chance alone. Alternately, our relatively high precurricular knowledge rates may have reflected a residency program already invested in addiction medicine teaching, with a robust addiction medicine consult service, an addiction medicine fellowship program, previously described AA[28] and brief intervention curricula,[27] and an addiction medicine elective. Additionally, education around substance use disorders is a public health priority, and several innovative curricula for medical students have been funded by the Association of American Medical Colleges[29] or highlighted in recent publications.[30] While the majority of these efforts focus specifically on prevention and treatment of opioid use disorder, rather than AUD, it is possible that increased attention to substance use disorders at the undergraduate medical level has led to reasonably high baseline medical knowledge. Importantly, however, residents themselves do not feel confident in their medical knowledge around MAUD, which is likely a key barrier to treating AUD in practice.

Our qualitative data hint at ongoing questions about logistical aspects of MAUD prescribing and the role of generalists in addiction management. While knowledge gaps are a key feature in attending reluctance to prescribe MAUD,[7,8] these data imply that further quality improvement efforts may be necessary to change practice patterns. Indeed, published quality improvement efforts to improve MAUD prescribing have had variable success; intensive one-on-one training with an individualized review of eligible patients improved prescribing rates,[31] while an emailed curriculum with access to local addiction experts did not.[32] Future studies of this curriculum could evaluate resident practice patterns and consider systems-level facilitators and barriers to AUD treatment in IM academic practices, including financial considerations, communication strategies for reluctant patients, attending practices, and formal partnerships with specialty addiction services.

In conclusion, our feasible curricular intervention for IM residents effectively increases knowledge, confidence, and attitudes in the management of AUD.

Appendices

A. Session 1 Learner Guide.docx
B. Session 1 Facilitator Guide.docx
C. Session 1 Concept Video.mp4
D. Session 2 Learner Guide.docx
E. Session 2 Facilitator Guide.docx
F. Session 2 Concept Video.mp4
G. Session 3 Learner Guide.docx

Copyright © 2022 Spinella et al. This is an open-access publication distributed under the terms of the Creative Commons Attribution license.

H. Session 3 Facilitator Guide.docx

I. Pre- and Postsurvey Tool.docx

J. Faculty Survey.docx

All appendices are peer reviewed as integral parts of the Original Publication.

Sara A. Spinella, MD, MS: Clinical Assistant Professor of Medicine, Department of Medicine, University of Pittsburgh School of Medicine and VA Pittsburgh Healthcare System; ORCID: https://orcid.org/0000-0002-1823-3959

Diana Samberg, MD, MS: Assistant Professor of Medicine, Department of Medicine, University of Pittsburgh School of Medicine

Melissa McNeil, MD, MPH: Professor of Medicine and Associate Chief of General Internal Medicine, Department of Medicine, University of Pittsburgh School of Medicine and VA Pittsburgh Healthcare System

Scott D. Rothenberger, PhD: Assistant Professor of Medicine and Statistician, Center for Research on Health Care Data Center, Department of Medicine, University of Pittsburgh School of Medicine

Payel Jhoom Roy, MD, MSc: Assistant Professor of Medicine and Clinical Director of Addiction Medicine Consult Service, Department of Medicine, University of Pittsburgh School of Medicine

Andrea E. Carter, MD, MS: Assistant Professor of Medicine and Associate Program Director of Internal Medicine Residency Program, Department of Medicine, University of Pittsburgh School of Medicine

Acknowledgments
We would like to acknowledge our funding sources: Veterans Health Administration Office of Academic Affiliations Advanced Fellowship in Women's Health and University of Pittsburgh Medical Center Department of General Internal Medicine.

Disclosures
None to report.

Funding/Support
Dr. Sara A. Spinella received funding from the Office of Academic Affiliations, Department of Veterans Affairs.

Prior Presentations
Spinella SA, Samberg D, McNeil M, Roy PJ, Carter AE. A video- and case-based curriculum on the management of alcohol use disorder for internal medicine residents during ambulatory pre-clinic conference. Poster presented virtually at: 2021 Society of General Internal Medicine; April 20, 2021.

Ethical Approval
The University of Pittsburgh and Veterans Affairs Pittsburgh Institutional Review Boards deemed further review of this project not necessary.

Disclaimer
The views expressed in this publication are our own and are not an official position of the Veterans Administration or the University of Pittsburgh.

References

1. Esser MB, Sherk A, Liu Y, et al. Deaths and years of potential life lost from excessive alcohol use—United States, 2011–2015. *MMWR Morb Mortal Wkly Rep*. 2020;69(39):1428-1433. https://doi.org/10.15585/mmwr.mm6939a6
2. Park-Lee E, Lipari RN, Hedden SL, Kroutil LA, Porter JD. Receipt of services for substance use and mental health issues among adults: results from the 2016 National Survey on Drug Use and Health. Updated September 2017. Accessed December 9, 2021. https://www.samhsa.gov/data/sites/default/files/NSDUH-DR-FFR2-2016/NSDUH-DR-FFR2-2016.htm
3. Haley SJ, Pinsker EA, Gerould H, Wisdom JP, Hagedorn HJ. Patient perspectives on alcohol use disorder pharmacotherapy and integration of treatment into primary care settings. *Subst Abus*. 2019;40(4):501-509. https://doi.org/10.1080/08897077.2019.1576089
4. Barry CL, Epstein AJ, Fiellin DA, Fraenkel L, Busch SH. Estimating demand for primary care-based treatment for substance and alcohol use disorders. *Addiction*. 2016;111(8):1376-1384. https://doi.org/10.1111/add.13364
5. Hepner KA, Paddock SM, Watkins KE, et al. Rates and impact of adherence to recommended care for unhealthy alcohol use. *J Gen Intern Med*. 2019;34(2):256-263. https://doi.org/10.1007/s11606-018-4749-3
6. Rittenberg A, Hines AL, Alvanzo AAH, Chander G. Correlates of alcohol use disorder pharmacotherapy receipt in medically insured patients. *Drug Alcohol Depend*. 2020;214:108174. https://doi.org/10.1016/j.drugalcdep.2020.108174
7. Harris AHS, Ellerbe L, Reeder RN, et al. Pharmacotherapy for alcohol dependence: perceived treatment barriers and action strategies among Veterans Health Administration service providers. *Psychol Serv*. 2013;10(4):410-419. https://doi.org/10.1037/a0030949
8. Williams EC, Achtmeyer CE, Young JP, et al. Barriers to and facilitators of alcohol use disorder pharmacotherapy in primary care: a qualitative study in five VA clinics. *J Gen Intern Med*. 2018;33(3):258-267. https://doi.org/10.1007/s11606-017-4202-z
9. Ehrie J, Hartwell EE, Morris PE, Mark TL, Kranzler HR. Survey of addiction specialists' use of medications to treat alcohol use disorder. *Front Psychiatry*. 2020;11:47. https://doi.org/10.3389/fpsyt.2020.00047

Copyright © 2022 Spinella et al. This is an open-access publication distributed under the terms of the Creative Commons Attribution license.

10. Tetrault JM, Green ML, Martino S, et al. Developing and implementing a multispecialty graduate medical education curriculum on screening, brief intervention, and referral to treatment (SBIRT). *Subst Abus*. 2012;33(2):168-181. https://doi.org/10.1080/08897077.2011.640220

11. Aurora P, Dumenco L, Mello MJ, George P. An interprofessional model for teaching medical students to provide screening, brief intervention, and referral for treatment for substance misuse. *R I Med J (2013)*. 2019;102(3):18-21.

12. Malone GP, Arismendez SV, Warzinski SS, et al. South Texas residency screening, brief intervention, and referral to treatment (SBIRT) training: 12-month outcomes. *Subst Abus*. 2015;36(3): 272-280. https://doi.org/10.1080/08897077.2014.988839

13. Stoner SA, Mikko AT, Carpenter KM. Web-based training for primary care providers on screening, brief intervention, and referral to treatment (SBIRT) for alcohol, tobacco, and other drugs. *J Subst Abuse Treat*. 2014;47(5):362-370. https://doi.org/10.1016/j.jsat.2014.06.009

14. Wamsley MA, Treit K, Satterfield JM, et al. Three standardized patient cases to measure screening, brief intervention and referral to treatment (SBIRT) skills in primary care residents. *MedEdPORTAL*. 2011;7:9005. https://doi.org/10.15766/mep_2374-8265.9005

15. Kahan M, Wilson L, Midmer D, Borsoi D, Martin D. Randomized controlled trial on the effects of a skills-based workshop on medical students' management of problem drinking and alcohol dependence. *Subst Abus*. 2003;24(1):5-16. https://doi.org/10.1080/08897070309511529

16. Fleming M, Olsen D, Stathes H, et al. Virtual reality skills training for health care professionals in alcohol screening and brief intervention. *J Am Board Fam Med*. 2009;22(4):387-398. https://doi.org/10.3122/jabfm.2009.04.080208

17. Pringle JL, Melczak M, Johnjulio W, Campopiano M, Gordon AJ, Costlow M. Pennsylvania SBIRT medical and residency training: developing, implementing, and evaluating an evidenced-based program. *Subst Abus*. 2012;33(3):292-297. https://doi.org/10.1080/08897077.2011.640091

18. Pringle JL, Kearney SM, Rickard-Aasen S, Campopiano MM, Gordon AJ. A statewide screening, brief intervention, and referral to treatment (SBIRT) curriculum for medical residents: differential implementation strategies in heterogeneous medical residency programs. *Subst Abus*. 2017;38(2):161-167. https://doi.org/10.1080/08897077.2017.1288195

19. Wamsley MA, Steiger S, Julian KA, et al. Teaching residents screening, brief intervention, and referral to treatment (SBIRT) skills for alcohol use: using chart-stimulated recall to assess curricular impact. *Subst Abus*. 2016;37(3):419-426. https://doi.org/10.1080/08897077.2015.1127869

20. Matassa D, Perrella B, Feurdean M. A novel team-based learning approach for an internal medicine residency: medication-assisted treatments for substance use disorders. *MedEdPORTAL*. 2021; 17:11085. https://doi.org/10.15766/mep_2374-8265.11085

21. Versteeg M, Hendriks RA, Thomas A, Ommering BWC, Steendijk P. Conceptualising spaced learning in health professions education: a scoping review. *Med Educ*. 2020;54(3):205-216. https://doi.org/10.1111/medu.14025

22. Roediger HL III, Agarwal PK, McDaniel MA, McDermott KB. Test-enhanced learning in the classroom: long-term improvements from quizzing. *J Exp Psychol Appl*. 2011;17(4):382-395. https://doi.org/10.1037/a0026252

23. Michael J. Where's the evidence that active learning works? *Adv Physiol Educ*. 2006;30(4):159-167. https://doi.org/10.1152/advan.00053.2006

24. van Houten-Schat MA, Berkhout JJ, van Dijk N, Endedijk MD, Jaarsma ADC, Diemers AD. Self-regulated learning in the clinical context: a systematic review. *Med Educ*. 2018;52(10):1008-1015. https://doi.org/10.1111/medu.13615

25. Thomas SE, Miller PM. Knowledge and attitudes about pharmacotherapy for alcoholism: a survey of counselors and administrators in community-based addiction treatment centres. *Alcohol Alcohol*. 2007;42(2):113-118. https://doi.org/10.1093/alcalc/agl100

26. Engs RC, Hanson DJ. The Student Alcohol Questionnaire: an updated reliability of the Drinking Patterns, Problems, Knowledge, and Attitude subscales. *Psychol Rep*. 1994;74(1):12-14. https://doi.org/10.2466/pr0.1994.74.1.12

27. Venkat A, Aldridge A, Kearney S, et al. Derivation of a shortened research instrument for measuring alcohol and other drug (AOD) attitudes in a screening, brief intervention, and referral to treatment (SBIRT) training program. *JOSHA*. 2017;4(2):290. https://doi.org/10.17160/josha.4.2.290

28. Kennedy AJ, McNeil M, Hamm M, Cameron FA, Carter AE. Internal medicine resident perceptions of patients with substance use disorder after attending a mutual support group meeting. *J Gen Intern Med*. 2020;35(3):918-921. https://doi.org/10.1007/s11606-019-05379-8

29. AAMC Curricular Innovation Awards. American Association of Medical Colleges. Accessed December 9, 2021. https://www.aamc.org/what-we-do/aamc-awards/curricular-innovation-awards

30. Pavuluri H, Poupore N, Schmidt WM, Boniface SG, Jindal M, Demosthenes L. Substance use disorder: a model for integration in undergraduate medical education. *J Med Educ Curric Dev*. 2021;8:23821205211025859. https://doi.org/10.1177/23821205211025859

31. Harris AHS, Bowe T, Hagedorn H, et al. Multifaceted academic detailing program to increase pharmacotherapy for alcohol use disorder: interrupted time series evaluation of effectiveness.

Copyright © 2022 Spinella et al. This is an open-access publication distributed under the terms of the Creative Commons Attribution license.

Addict Sci Clin Pract. 2016;11:15. https://doi.org/10.1186/s13722-016-0063-8

32. Harris AHS, Brown R, Dawes M, et al. Effects of a multifaceted implementation intervention to increase utilization of pharmacological treatments for alcohol use disorders in the US Veterans Health Administration. *J Subst Abuse Treat*. 2017;82:107-112. https://doi.org/10.1016/j.jsat.2017.09.002

Received: July 3, 2021
Accepted: January 8, 2022
Published: March 31, 2022

Copyright © 2022 Spinella et al. This is an open-access publication distributed under the terms of the Creative Commons Attribution license.
